# Resuscitation dynamics reveal persister partitioning after antibiotic treatment

**DOI:** 10.15252/msb.202211320

**Published:** 2023-03-03

**Authors:** Xin Fang, Kyle R Allison

**Affiliations:** ^1^ Wallace H. Coulter Department of Biomedical Engineering Emory University and Georgia Institute of Technology Atlanta GA USA; ^2^ Department of Medicine/Division of Infectious Diseases Emory University School of Medicine Atlanta GA USA

**Keywords:** antibiotics, dynamics, partitioning, persister, resuscitation, Microbiology, Virology & Host Pathogen Interaction

## Abstract

Bacteria can survive antibiotics by forming dormant, drug‐tolerant persisters. Persisters can resuscitate from dormancy after treatment and prolong infections. Resuscitation is thought to occur stochastically, but its transient, single‐cell nature makes it difficult to investigate. We tracked the resuscitation of individual persisters by microscopy after ampicillin treatment and, by characterizing their dynamics, discovered that *Escherichia coli* and *Salmonella enterica* persisters resuscitate exponentially rather than stochastically. We demonstrated that the key parameters controlling resuscitation map to the ampicillin concentration during treatment and efflux during resuscitation. Consistently, we observed many persister progeny have structural defects and transcriptional responses indicative of cellular damage, for both β‐lactam and quinolone antibiotics. During resuscitation, damaged persisters partition unevenly, generating both healthy daughter cells and defective ones. This persister partitioning phenomenon was observed in *S. enterica*, *Klebsiella pneumoniae*, *Pseudomonas aeruginosa*, and an *E. coli* urinary tract infection (UTI) isolate. It was also observed in the standard persister assay and after *in situ* treatment of a clinical UTI sample. This study reveals novel properties of resuscitation and indicates that persister partitioning may be a survival strategy in bacteria that lack genetic resistance.

## Graphical abstract

Tracking and modelling single‐cell resuscitation dynamics of bacterial persisters shows that it is a drug‐dependent and non‐stochastic process. Persisters can partition cellular damage into healthy and nonviable daughter cells after antibiotic treatment.

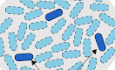

## Introduction

Bacterial persisters survive antibiotics by temporarily entering a nondividing state through multiple possible formation mechanisms (Lewis, 2007; Cohen *et al*, 2013; Orman & Brynildsen, 2015; Dewachter *et al*, 2019; Gollan *et al*, 2019; Kaldalu *et al*, 2020). As the lethality of most antibiotics requires cellular growth, dormancy may provide a broad‐spectrum tolerance strategy by blocking drug uptake, preventing target binding (Lewis, 2007), or by reducing the lethal downstream consequences of antibiotics (Allison *et al*, 2011a). Genetic resistance is therefore not required for persisters to survive treatment, after which they can resuscitate and contribute to chronic and recurrent infections. Understanding resuscitation, which returns persisters to antibiotic susceptibility, could lead to improved use of currently existing antibiotics. However, its transient single‐cell nature makes it difficult to investigate. Recent studies exploring resuscitation suggest important roles for toxins, DNA‐repair systems, ribosome rescue, and even chemotaxis (Volzing & Brynildsen, 2015; Mok & Brynildsen, 2018; Wilmaerts *et al*, 2019; Mohiuddin *et al*, 2020, 2022; Yamasaki *et al*, 2020), but key aspects remain unclear including resuscitation's dynamics and the parameters controlling it.

Dynamical modeling of individual cells can provide a useful framework for understanding transient behaviors (Alon *et al*, 1999; Elowitz *et al*, 2002; Gefen *et al*, 2008; Luidalepp *et al*, 2011; Aldridge *et al*, 2012; Wakamoto *et al*, 2013; Joers & Tenson, 2016; Simsek & Kim, 2019; Basan *et al*, 2020; Moreno‐Gámez *et al*, 2020). Bulk population dynamics and the cellular lag times after nutrient limitation (Keren *et al*, 2004; Luidalepp *et al*, 2011; Joers & Tenson, 2016; Gutierrez *et al*, 2017; Vulin *et al*, 2018; Zheng *et al*, 2020; Manuse *et al*, 2021) suggest persister resuscitation might occur stochastically (Balaban *et al*, 2004; Kaplan *et al*, 2021), meaning persisters randomly “wake up.” Such stochasticity could arise from the reversal of persister formation mechanisms, as suggested with toxin–antitoxin systems (Rotem *et al*, 2010) or from network effects of cellular aging (Kaplan *et al*, 2021). The stochastic model suggests persistence is a probabilistic “bet‐hedging” strategy and that resuscitation occurs independently of antibiotics (Kussell *et al*, 2005; Kussell & Leibler, 2005), causing cell death when it takes place during treatment. Although these ideas are supported by the available evidence, they have not yet been confirmed in individual persister cells. Hence, we developed approaches to track resuscitation directly and then mathematically model the resulting dynamics. This “bottom‐up” approach to resuscitation was agnostic to the underlying molecular mechanisms and therefore suited to provide general insights.

By single‐cell tracking, we found that ampicillin *E. coli* persisters resuscitate exponentially rather than stochastically. After treatment, resuscitation is initially slow but accelerates, raising the possibility it is delayed by antibiotic treatment and may rely on efflux. We validate these model predictions, showing that the parameters determining resuscitation map to antibiotic concentration during the treatment phase and to efflux ability during the resuscitation phase. We further observe that the resuscitated progeny of many persisters have structural defects and transcription indicative of antibiotic damage, for both β‐lactams and quinolones. Relatedly, we identify distinct cell‐fate trajectories in each resuscitating population: healthy, damaged, and failed persisters. The type of damage observed in persister progeny is determined by the treatment antibiotic's mode of action. We found that cell division in damaged persisters simultaneously produces both healthy daughter cells and nonviable ones. We showed that this persister partitioning is a general aspect of resuscitation in multiple species and in response to multiple antibiotics. Moreover, we demonstrate persister partitioning occurs after antibiotic treatment in the standard persister assay and *in situ* in a sample taken directly from a patient‐urinary‐tract infection. Our findings indicate that resuscitation is a drug‐responsive (rather than drug‐independent) phase of persistence that utilizes efflux and uneven cellular partitioning for survival.

## Results

### Single‐cell persister resuscitation reveals two‐state model dynamics

**Figure 1:**
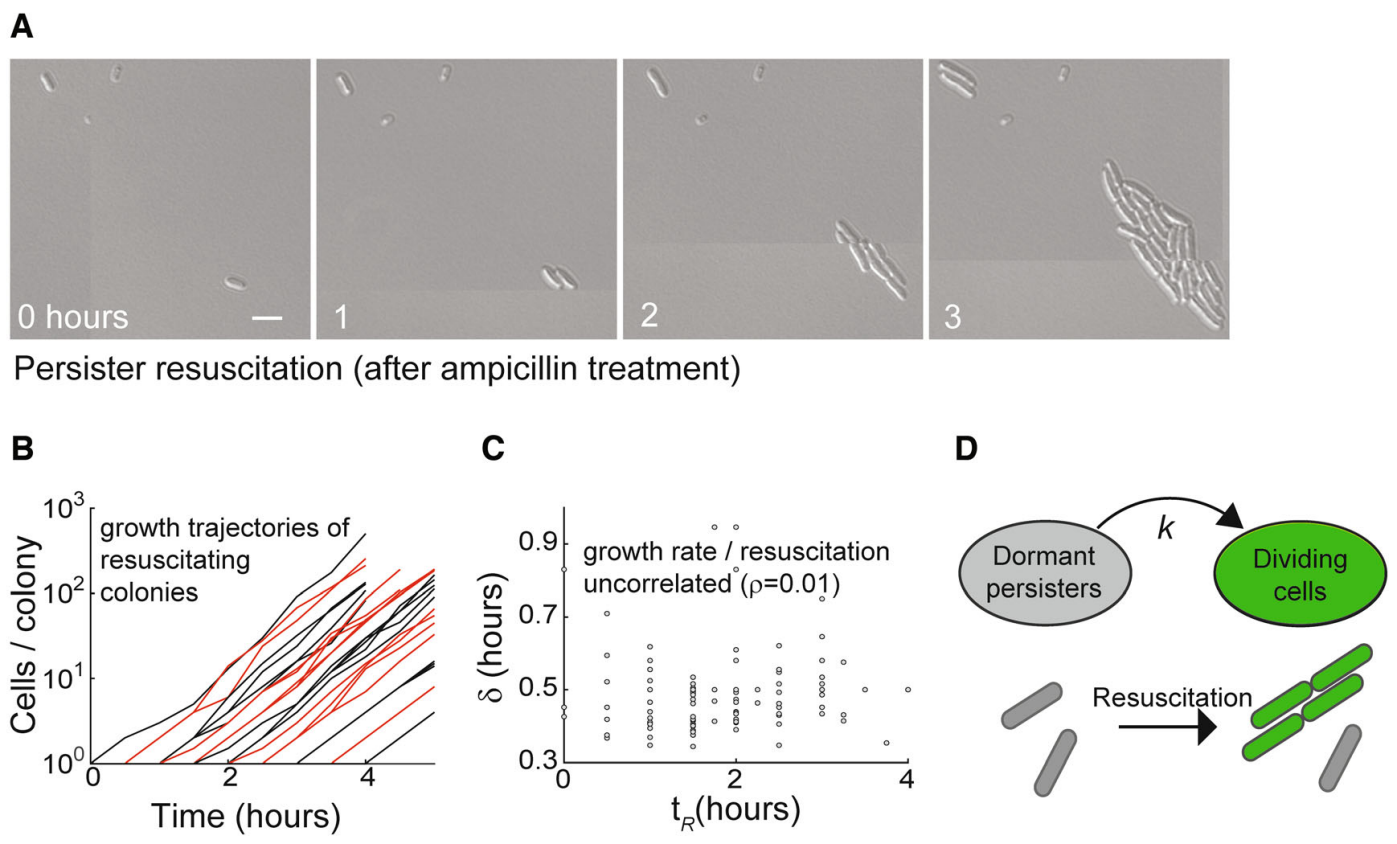
Monitoring persister resuscitation at the single‐cell level Example micrographs of single‐cell persister resuscitation after ampicillin (100 μg/ml) treatment. Tiled images have been acquired and stitched. Scale bar: 2 μm.Growth of persister progeny after resuscitation (illustrative data for 27 colonies, lineages are alternatingly colored black and red).Cellular doubling time (δ) of persister progeny as a function of resuscitation time (*t*_*R*_) (*n* = 127 cell lineages). Correlation coefficient (*ρ*) is indicated on plot.Dynamical model of resuscitation as a two‐state process governed by rate parameter (*k*).

We used single‐cell time‐lapse microscopy to track resuscitation in individual persisters and model their dynamics. Stationary‐phase *E. coli* were added to fresh media and treated with β‐lactam antibiotic ampicillin (see Materials and Methods) which killed over 99% of cells within 3 hours (Appendix Fig S1). The remaining intact cells were thoroughly washed to remove antibiotics and then incubated on agarose slides, which allowed us to distinguish the trajectories of persisters as they formed separate microcolonies. Resuscitation was monitored by imaging cells every 30 min (Fig 1A). The resuscitation time (*t*_*R*_, time of first cell division) and cellular doubling time (*δ*) for each resuscitated persister (228 individual lineages) were quantified. After resuscitation, cells grew at a consistent rate (Fig 1B) which was uncorrelated with the time they had resuscitated (Fig 1C). This suggests that resuscitation dynamics after ampicillin treatment can be effectively modeled as a simple transition between dormant and dividing states (Fig 1D).

### Persister resuscitation rate is exponential

**Figure 2:**
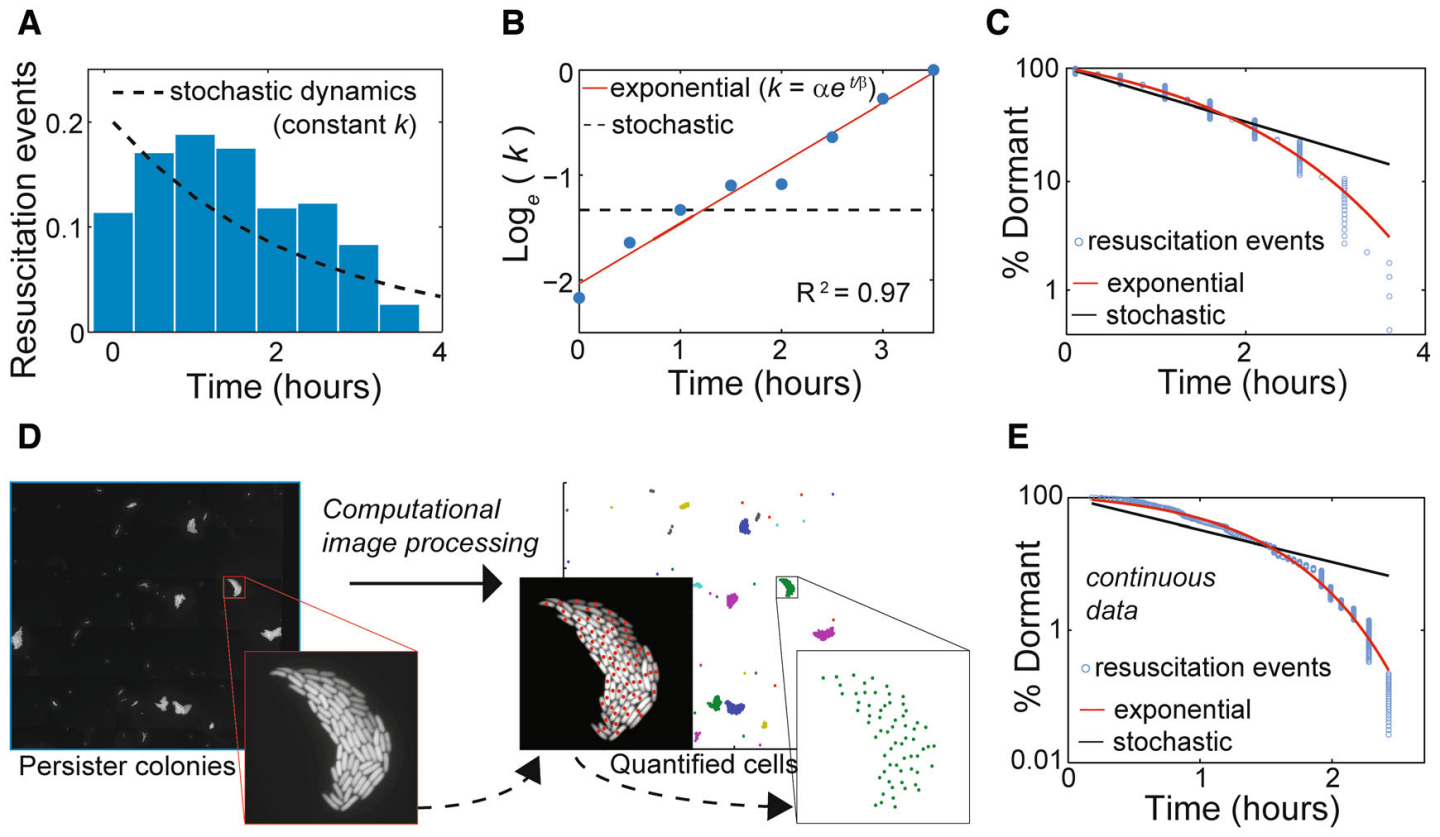
Exponential persister resuscitation Normalized distribution of *t*_*R*_ (*n* = 228 cell lineages), with stochastic model fit (dashed line).Time‐dependence of *k* calculated from data in (A). Exponential fit ($k=\alpha e^{\mathrm{\beta t}}$, red line) with *R* ^2^ and stochastic fit (dashed black line) are depicted.Resuscitation curve (% persisters still dormant as a function of time) with stochastic (dashed black line) and exponential (red line) model fits.Illustration of automated image analysis for fluorescence experiments to determine continuous‐time *t*_*R*_ values. Tiled images have been acquired and stitched. The same representative image is used in Appendix Fig S5.Resuscitation curve based continuous‐time *t*_*R*_, with stochastic (dashed black line) and exponential (red line) model fits (*n* = 645 microcolonies).

Persistence has previously been described by a two‐state model using differential equations, which suggest persisters resume growth stochastically (Balaban *et al*, 2004; Kaplan *et al*, 2021):

(1) $$\frac{\mathrm{dP}}{\mathrm{dt}}=-\mathrm{kP},$$

where *P* is number of persisters and *k* is a rate constant. The number of persisters as a function of time can be determined by integrating equation (1):

(2) $$P_{t}=e^{-\mathrm{kt}},$$

in which *P*_*t*_ is the ratio of persisters yet to resuscitate. However, the observed resuscitation dynamics did not fit this model (Fig 2A). Instead, the rate of resuscitation accelerated (Appendix Fig S2) and was not constant as indicated by the stochastic model. To assess the temporal dependence of this transition, we calculated *k* on 30‐min intervals and found that it increased exponentially with time (Fig 2B), indicating:

(3) $$\frac{\mathrm{dP}}{\mathrm{dt}}=\alpha e^{\mathrm{\beta t}}P,$$

where *α* and *β* are empirical parameters that may correspond to underlying determinants of resuscitation. Including this relationship for *k* into equation (1), we derived a model that fit the observed resuscitation dynamics (Fig 2C):

(4) $$P_{t}=e^{\alpha /\beta \left(e^{\mathrm{\beta t}}-1\right)}$$

Applying approaches from structural equations modeling (Bollen, 1989; Kline, 2015), we statistically compared the stochastic and exponential models (equations 2 and 4). Both residual analysis (Appendix Fig S3) and the nested‐model χ^2^‐difference test indicated a preference for the exponential model (equation 4), though the significance of χ^2^‐difference test (*P*‐value, 0.077) was not sufficient to confidently reject the stochastic model (equation 2). This indicated additional evidence was needed for statistical certainty. A limitation of the collected data was the 30‐min sampling interval of the experiments which did not allow for precise, continuous‐time determinations of *t*_*R*_ values. To overcome this, we imputed *t*_*R*_ (Appendix Fig S4) from the number of cells in persister‐derived microcolonies using the growth kinetics of persister progeny (see Fig 1B and C):

(5) $$t_{R}=t-\delta \;\log_{2}\left(N_{t}\right),$$

where *N*_*t*_ is the number of cells at time *t*. We used a wild‐type strain that constitutively expressed green fluorescent protein to automate computational image processing (Fig 2D and Appendix Fig S5) and calculation of *t*_*R*_ from equation (5). Variance in growth rate should introduce noise rather than bias in *t*_*R*_ values, as *δ* an *t*_*R*_ are uncorrelated (Fig 1C). Resulting *t*_*R*_ values (from 645 microcolonies) fit exponential dynamics (Fig 2E and Appendix Fig S6) and the nested‐model χ^2^‐difference method demonstrated that equation (4) is strongly preferable to equation (2) (*P*‐value, 2.6 × 10^−7^). These results show resuscitation of ampicillin persisters occurs exponentially. As a control, we verified that imputed values correlated with directly observed resuscitation times (Appendix Fig S7). Persister resuscitation dynamics were found to be independent of cell density (Appendix Fig S8), suggesting a key difference from stationary phase resuscitation which depends on available muropeptides (Joers *et al*, 2019). Importantly, the exponential and stochastic models would be empirically equivalent when fit to bulk‐scale or colony‐appearance‐time data, and our ability to distinguish these models resulted from capturing the initial stage of resuscitation at the cellular scale. In the future, further data precision or scale might support more complicated dynamics and refine the exponential model.

Equations (2) and (4) have different implications, though they are quantitatively and formally similar. The stochastic model (equation 2) indicates resuscitation is antibiotic‐independent and may result from reversing the formation mechanisms (Korch *et al*, 2003; Balaban *et al*, 2004; Rotem *et al*, 2010). The exponential model (equation 4) indicates resuscitation is initiated by the end of treatment and may be in part independent of persister formation mechanisms. In practice, a model's parameters must correspond to underlying molecular determinants. Hence, the parameters in equation (4) are predicted to be dependent on ampicillin. Relatedly, equation (4)'s structure implies that resuscitation contains a form of positive feedback:

(6) $$\frac{\mathrm{dk}}{\mathrm{dt}}=f\left(k\right),$$

though the precise mechanism will require further investigation. The independence of formation and resuscitation, the role of antibiotics, and the role of positive‐feedback are all testable implications unique to the exponential model (equation 4).

### Persister resuscitation in *hipA7 E. coli* and *S. enterica* is exponential

We sought to investigate the generality of exponential dynamics and, simultaneously, if different formation mechanisms produced different resuscitation dynamics. The high‐persistence *E. coli* strain (*hipA7*) has two gain‐of‐function mutations in the *hipA* toxin gene (Korch *et al*, 2003; Germain *et al*, 2013; Semanjski *et al*, 2018) and is commonly used as a model system for persistence. It represents a separate formation mechanism from that of wild‐type that can boost persister numbers by orders of magnitude. Applying the fluorescent‐based approach (Fig 2D), we calculated *t*_*R*_ values for *hipA7* persisters and found these cells also resuscitated exponentially (Appendix Figs S9 and S10). Quantitatively, *hipA7* resuscitation was indistinguishable from wild‐type, demonstrating that resuscitation dynamics were consistent despite differing formation mechanisms. Further extending this analysis, we investigated the dynamics in *Salmonella enterica*, a clinically important bacterium that forms persisters via differing mechanisms (Cheverton *et al*, 2016; Fisher *et al*, 2017; Pontes & Groisman, 2019). *Salmonella enterica* persisters also resuscitated exponentially after treatment (Appendix Fig S11). These findings demonstrate a surprising commonality in resuscitation dynamics and indicate that resuscitation is, in part, separate from persister formation mechanisms.

### Antibiotic treatment delays persister resuscitation

**Figure 3:**
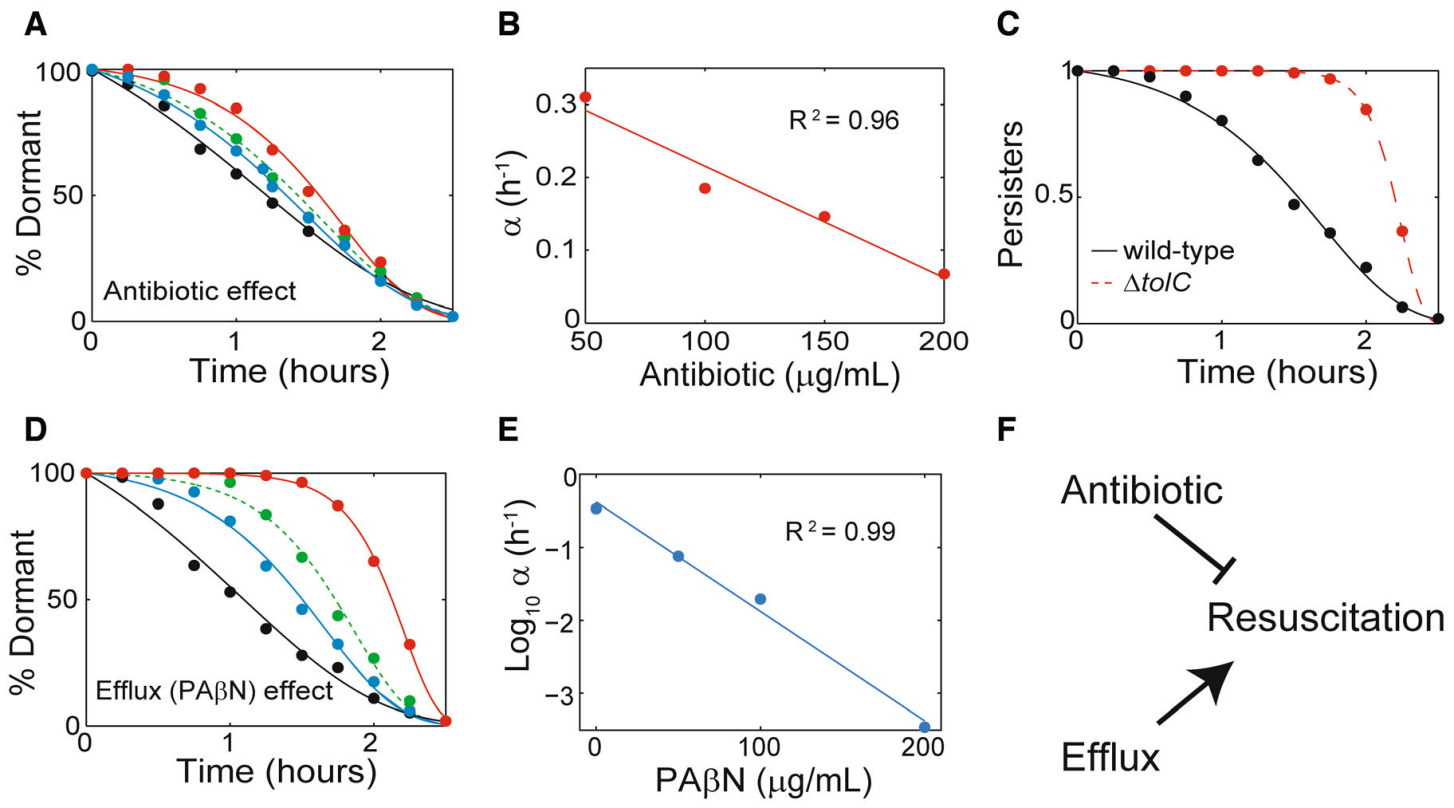
Parameters controlling persister resuscitation Resuscitation curves after cells were treated with different antibiotic (ampicillin) concentrations (black, 50 μg/ml, *n* = 279; blue, 100 μg/ml, *n* = 261; green, 150 μg/ml, *n* = 214; and red 200 μg/ml, *n* = 178. Data were binned into 10 time points). *t*_*R*_ values calculated with continuous‐time approach; curves depict exponential‐model fits.Resuscitation parameter α dependence on antibiotic treatment concentration, calculated from data presented in (A).Resuscitation curve for wild‐type (BW25113; *n* = 330) and efflux‐impaired Δ*tolC* (*n* = 122) strains with exponential‐model fit curves.Resuscitation curves for persisters incubated with different efflux inhibitor (PAβN) concentrations (black, 0 μg/ml, *n* = 121; blue, 50 μg/ml, *n* = 171; green, 100 μg/ml, *n* = 186; and red 200 μg/ml, *n* = 107) with exponential‐model fit curves.Resuscitation parameter α dependence on PAβN concentration, calculated from data presented in (D).Schematic representation of resuscitation dynamics, controlled by antibiotic concentration during treatment and molecular efflux after treatment.

We next explored the predicted role of antibiotics in resuscitation. We studied the resuscitation of cells treated with antibiotics for different periods of time (Appendix Fig S12). Persisters treated for 4.5 and 6 h had identical resuscitation dynamics. Persisters treated for 3 h resuscitated marginally faster, though those treated for 12 h were substantially slower. This indicated that, in most cases, resuscitation rate is not strongly dependent on the duration of treatment. We also investigated whether the antibiotic concentration during the treatment phase affected resuscitation dynamics. Persisters that had been treated with higher concentrations were slower to resuscitate (Fig 3A and Appendix Fig S13), demonstrating that antibiotic treatment delays resuscitation. This further suggested that the concentration of the treatment phase might determine the parameters in equation (4). To test this, we plotted antibiotic concentration and corresponding values for *α* which revealed a linear, negative relationship between the experimental parameter and the mathematical one (*R* ^2^ = 0.96; Fig 3B). Thus, a simple relationship exists between ampicillin concentration during treatment and resuscitation rate after treatment. This may suggest the possibility that treatment impedes resuscitation, an idea suggested by Joseph Bigger in his original identification of persisters (Bigger, 1944) and anticipated as a possible alternative to the stochastic model (Kussell & Leibler, 2005).

### Efflux facilitates persister resuscitation

The effect of antibiotics led us to hypothesize that resuscitation may require efflux, the homeostatic process that detoxifies intracellular antibiotics by coupling inner‐membrane ABC transporters with the outer‐membrane protein *tolC* (Koronakis *et al*, 2004; Delmar *et al*, 2014; Bergmiller *et al*, 2017; Du *et al*, 2018). Persister resuscitation in a Δ*tolC* mutant, in which antibiotic efflux is impaired, was strongly delayed (Fig 3C and Appendix Fig S14), demonstrating that efflux is essential to resuscitation. The effect of Δ*tolC* could be through blocking efflux during the resuscitation phase or alternatively by increasing ampicillin uptake during the treatment phase, thereby delaying resuscitation in a drug‐dependent manner (Fig 3A and B). To distinguish between these possibilities, we used Phe‐Arg‐β‐naphthylamide (PAβN), a small molecule that inhibits efflux without affecting normal growth (Matsumoto *et al*, 2011). We performed resuscitation experiments with wild‐type in which PAβN was added only during resuscitation and therefore could not affect the uptake of antibiotic during treatment. We found that PAβN delayed resuscitation in a concentration‐dependent manner (Fig 3D and Appendix Fig S15), demonstrating the role of efflux during resuscitation. PAβN did not affect the total number of persisters that resuscitated, though it delayed colony‐appearance‐times (Appendix Fig S16). Values of *α* and PAβN concentration revealed an exponential relationship between the two parameters (*R* ^2^ = 0.99; Fig 3E). This exponential scaling might suggest that efflux contributes to the positive‐feedback loop inherent in the resuscitation dynamics. A possible but untested explanation is as follows: Efflux enables cellular growth which in turn increases the requisite transporters and energy for further efflux and growth. Of note, persister levels of wild‐type and Δ*tolC* strains were comparable, suggesting efflux plays a minor role in the formation and treatment phases of persistence (Appendix Fig S17). As a caveat, Δ*tolC* and PAβN both delayed stationary phase resuscitation though to a lesser degree than in persister (Appendix Fig S18). This may indicate an additional role in resuscitation separate from antibiotic efflux. Our findings demonstrate the critical role of efflux to persister resuscitation (Fig 3F), but also indicate further studies are needed to precisely define this role.

### Structural damage in ampicillin persisters

**Figure 4:**
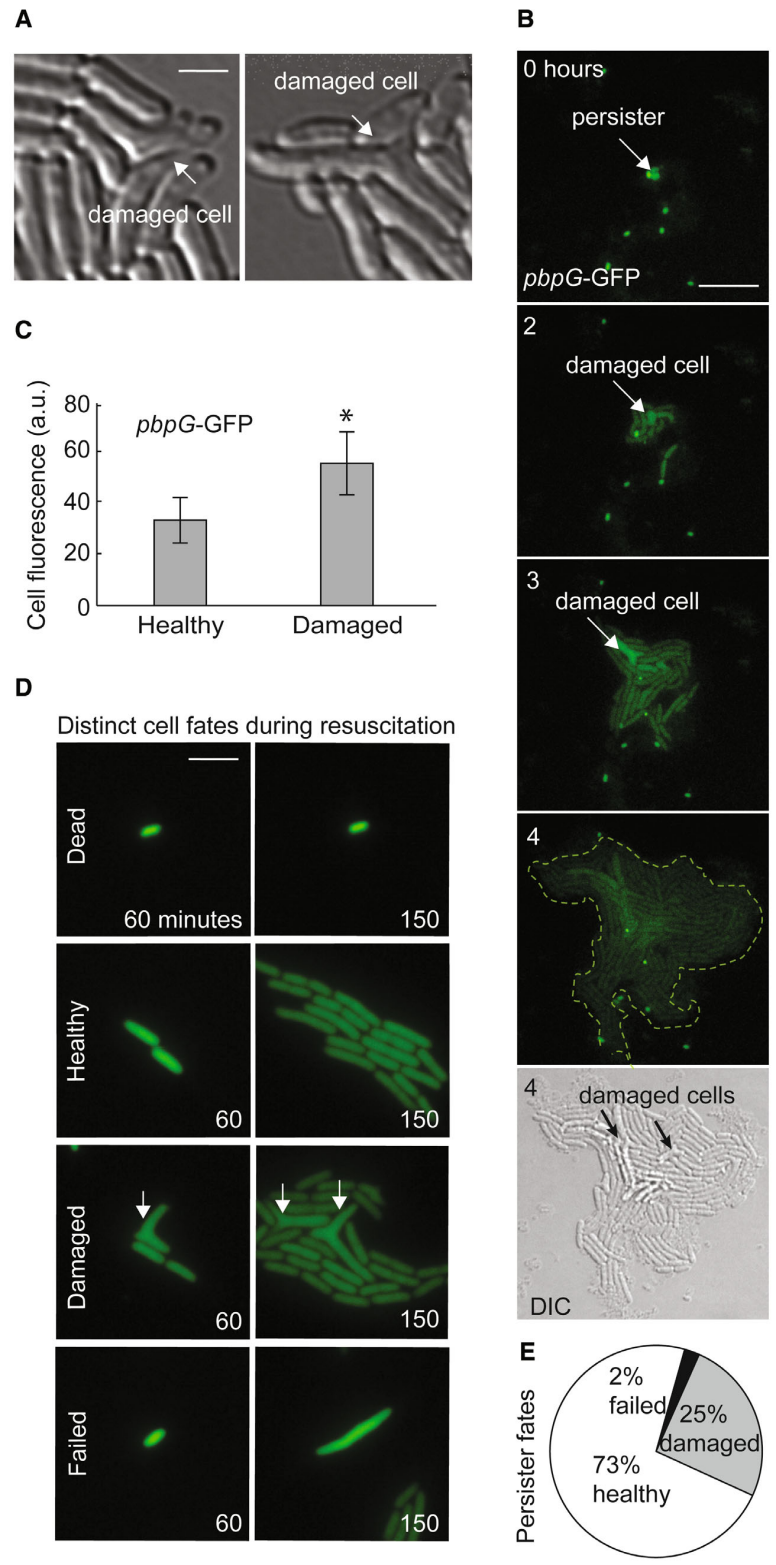
Ampicillin damage in persister progeny Example micrographs of progeny derived from a single persister after resuscitation from ampicillin treatment. Cell structural damage is indicated by white arrow. Scale bar: 2 μm.Representative example of resuscitation in persister carrying fluorescent transcriptional reporter *pbpG*‐GFP. Scale bar: 8 μm.Fluorescence of healthy and damaged persister progeny cells containing *pbpG*‐GFP 2 h after the end of ampicillin treatment (values represent mean ± SD, *n* = 12, biological replicates; * indicates *P* < 0.01 by Student's *t* test).Representative examples of the distinct resuscitation fates of individual cells observed after ampicillin treatment. Cells contained constitutive *ompC*‐GFP for visualization. Arrows indicate damaged cells. Scale bar: 5 μm.Pie chart depicting the relative proportions of healthy, damaged, and failed persisters after ampicillin treatment. Results calculated from four data sets.

The role of antibiotics and efflux in resuscitation suggested to us that ampicillin may directly damage persisters. To explore this, we inspected the resuscitated persister progeny within microcolonies, each deriving from a single persister cell. We found that some microcolonies contained cells with structural defects indicative of drug‐target binding (Fig 4A and Appendix Fig S19). The observed structural defects were not visible in cells prior to resuscitation and occurred in 1–8 cells within individual microcolonies, suggesting that an individual persister could divide into both healthy and damaged daughter cells. These defects are consistent with perturbations to ampicillin's molecular target, the penicillin‐binding proteins (PBPs) that determine cell shape. However, they are distinct from ampicillin's effect on normal susceptible cells which form bulges and lyse (Yao *et al*, 2012). Currently, the only known cause of the observed structural defects is depletion of PBPs (de Pedro *et al*, 2003; Potluri *et al*, 2012). To further test this, we investigated resuscitation in cells harboring a fluorescent transcriptional reporter for *pbpG* (encoding PBP7). We observed that damaged cells had higher transcription form this promoter (Fig 4B and C, and Appendix Figs S20 and S21), further suggesting these defects may have resulted from PBP depletion.

### Ampicillin persisters resuscitate with diverse phenotypes

To further characterize the cellular phenotypes during resuscitation, we recorded videos tracking hundreds of individual persisters as they resuscitated and formed separate microcolonies (Dataset EV1: Videos A1–A6). We observed distinct persister‐cell‐fate trajectories which we label as follows: dead cells, healthy (undamaged) persisters, damaged persisters, and failed persisters (Fig 4D). Dead cells had no cellular growth and represented the majority of cells visible by microscopy. The three “persister” phenotypes displayed cellular growth after ampicillin treatment, but differed in cellular structure and ability to successfully complete cell division. Healthy persisters produced progeny resembling normally growing cells and had no visible defects. They comprised ~73% of the persister population. Damaged persisters displayed triangular/branching defects consistent with ampicillin‐target binding and comprised ~25% of the persister population (Fig 4E) and commonly had higher *pbpG* induction (Appendix Fig S22 and Dataset EV1: Videos B1–B3). Failed persisters initially resembled damaged persisters and had cellular growth, but ultimately did not complete cell division and did not produce colonies (Appendix Fig S23). They ceased cellular growth and often lost membrane integrity, evidenced by a sudden density shift in DIC imaging or loss of signal in fluorescent imaging, and comprised ~2% of the persister population. Similar experiments in the BW25113 strain revealed the persister population was composed of 79% healthy, 20% damaged, and 1% failed persisters (Appendix Fig S24). Treatment with higher concentrations of ampicillin did not significantly change the cell‐fate distributions (Appendix Fig S25). These findings indicate β‐lactam persisters can follow different cell‐fate trajectories during resuscitation.

### Persister partitioning after β‐lactam treatment

**Figure 5:**
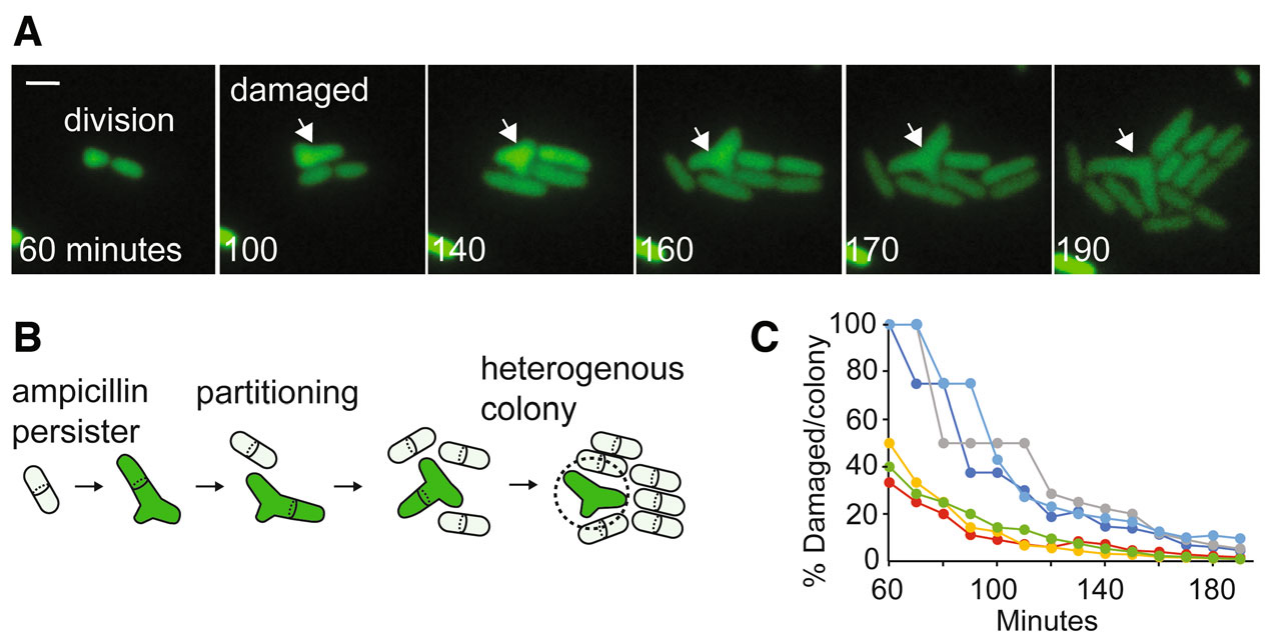
Persister partitioning after ampicillin treatment Representative example of resuscitation and partitioning in a persister after ampicillin treatment. Cells contained constitutive *ompC*‐GFP for visualization. 1^st^ division is shown in 60‐min image and arrows indicate damaged cells. Scale bar: 2 μm.Diagram illustrating the observed ampicillin persister partitioning during resuscitation.Percentage of progeny with structural defects in microcolonies derived from damaged ampicillin persisters. Data obtained from six representative microcolonies.

We further investigated the trajectories of damaged persisters and observed that they divided to create both viable cells that grew normally and damaged cells that grew slowly (Fig 5A). Hence, damage in some daughter cells is permanent and can be observed in microcolonies hours after the initial persister resuscitation event (Appendix Fig S26). To our knowledge, such partitioning of persister cells after ampicillin treatment has not been previously reported (Fig 5B). We hypothesized that damaged cells would rapidly be outnumbered in resuscitated populations, and calculated the frequency of damaged cells within individual persister microcolonies as a function of time. We found that the frequency of damaged cells within microcolonies declines to less than 1% of cells within hours of resuscitation due to the exponential growth of healthy sister cells (Fig 5C). This result indicates that damaged cells are only transiently observable after resuscitation in most experiments, and in part explains why persister partitioning has not been previously reported. The precise contribution of persister partitioning to β‐lactam survival warrants further investigation, though it evidently allows a persister to create one healthy daughter cell rather than fail to resuscitate due to damage.

### Persister partitioning after quinolone treatment

**Figure 6:**
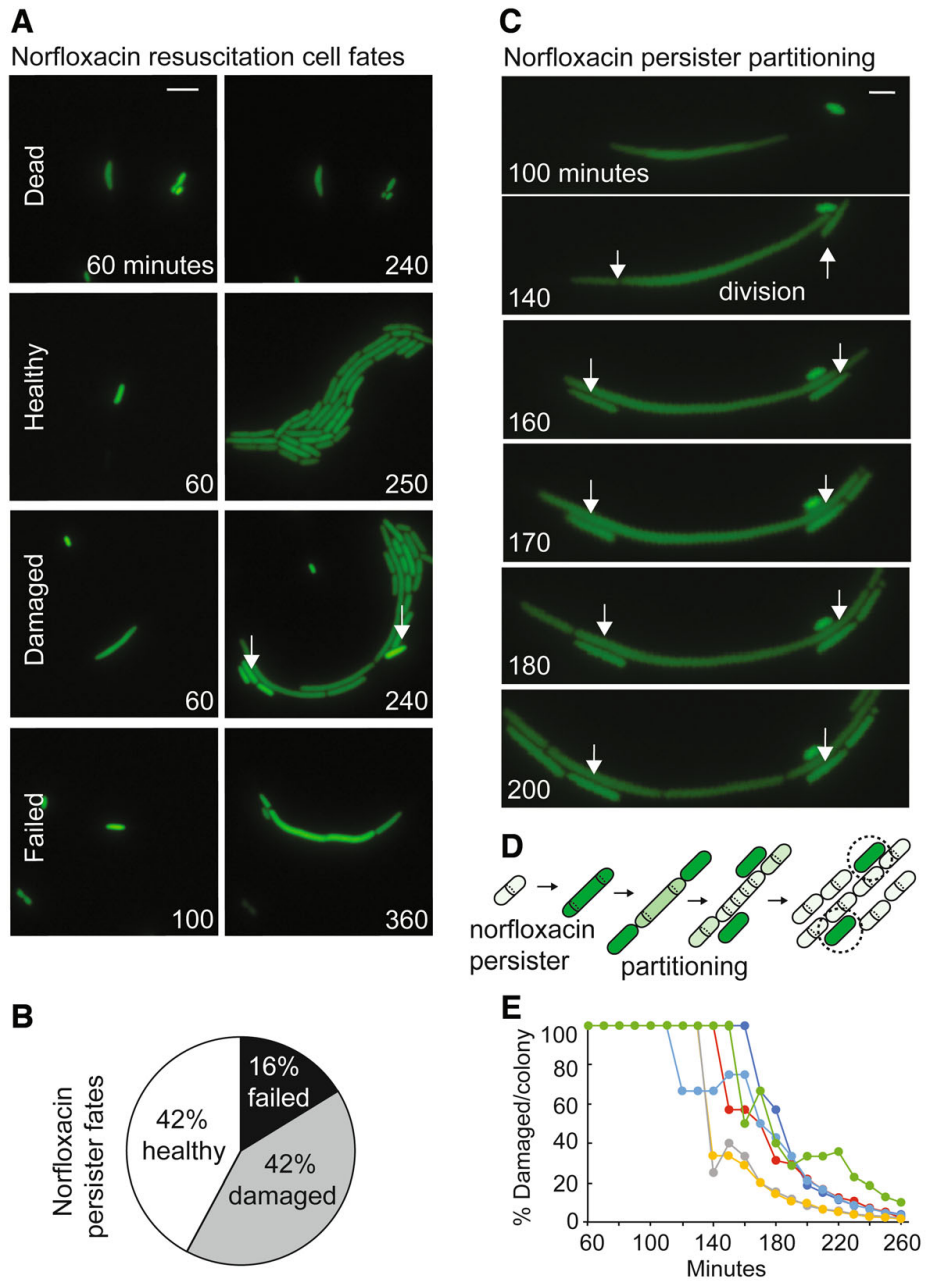
Distinct resuscitation fates and persister partitioning after norfloxacin treatment Representative example of the resuscitation fates of individual cells observed after norfloxacin treatment. Cells contained constitutive *ompC*‐GFP for visualization. Arrows indicate non‐growing persister progeny cells. Scale bar: 5 μm.Pie chart depicting the relative proportions of healthy, damaged, and failed persisters after norfloxacin treatment. Results calculated from three data sets. The same figure is used as wild‐type control in Appendix Fig S32.Representative example of resuscitation and partitioning in a persister after norfloxacin treatment. Cells contained constitutive *ompC*‐GFP for visualization. 1^st^ and 2^nd^ divisions occur at the 140‐min image and arrows indicate nongrowing persister progeny cells. Scale bar: 2 μm.Diagram illustrating the observed norfloxacin persister partitioning during resuscitation.Percentage of damaged/nongrowing progeny in microcolonies derived from damaged norfloxacin persisters. Data obtained from six representative microcolonies.

Given recent mechanistic insights (Volzing & Brynildsen, 2015; Mok & Brynildsen, 2018; Barrett *et al*, 2019), we reasoned persister partitioning might also occur after treatment with quinolone antibiotics, which target DNA remodeling enzymes (Rehrauer *et al*, 1996). Hence, we tracked resuscitation of quinolone persisters at the single‐cell level (Dataset EV1: Videos C1–C6). After treatment, the majority visible cells are dead and most persisters (90%) filamented during resuscitation, a result of the SOS response blocking septation (Huisman *et al*, 1980; Dopazo *et al*, 1987) after DNA damage. A separate mathematical model will be required to study quinolone resuscitation dynamics in the future, but given the cellular filamentation and long delay time before first division, neither stochastic nor exponential models will provide reasonable fits. As seen in ampicillin persisters, many quinolone persisters produced both viable and nonviable daughter cells (Fig 6A). Therefore, for quinolones we defined “healthy” persisters as those that produced only normally growing progeny and “damaged” persisters as those that produced both growing and nongrowing progeny. Healthy (42%) and damaged (42%) persisters accounted for similar portions on the population (Fig 6B). Failed quinolone persisters grew and filamented (Appendix Fig S27), but similar to ampicillin, they did not divide and often had a sudden loss of fluorescence that may indicate loss of membrane integrity. Failed persisters comprised ~16% of the quinolone persister population. These observations demonstrate that quinolone persisters also have different cell fates during resuscitation, including persisters that partition into both viable and nonviable daughter cells. Treatment with higher concentrations of quinolone did not significantly change the cell‐fate distributions (Appendix Fig S28). In the damaged cells, we observed that the first cell division produced nongrowing cells from the parental poles of the filamented persisters (Fig 6C). Subsequent, pole proximal division events often produced further nonviable cells after the first division, before the more central cites on the cell divided and produced healthy daughter cells. To our knowledge, such partitioning of persister cells after norfloxacin treatment has not been previously reported (Fig 6D). Similar to ampicillin, the frequency of nonviable daughter cells within microcolonies declined to less than 1% of cells within hours after norfloxacin resuscitation (Fig 6E).

### Quinolone persister partitioning requires SOS response

**Figure 7:**
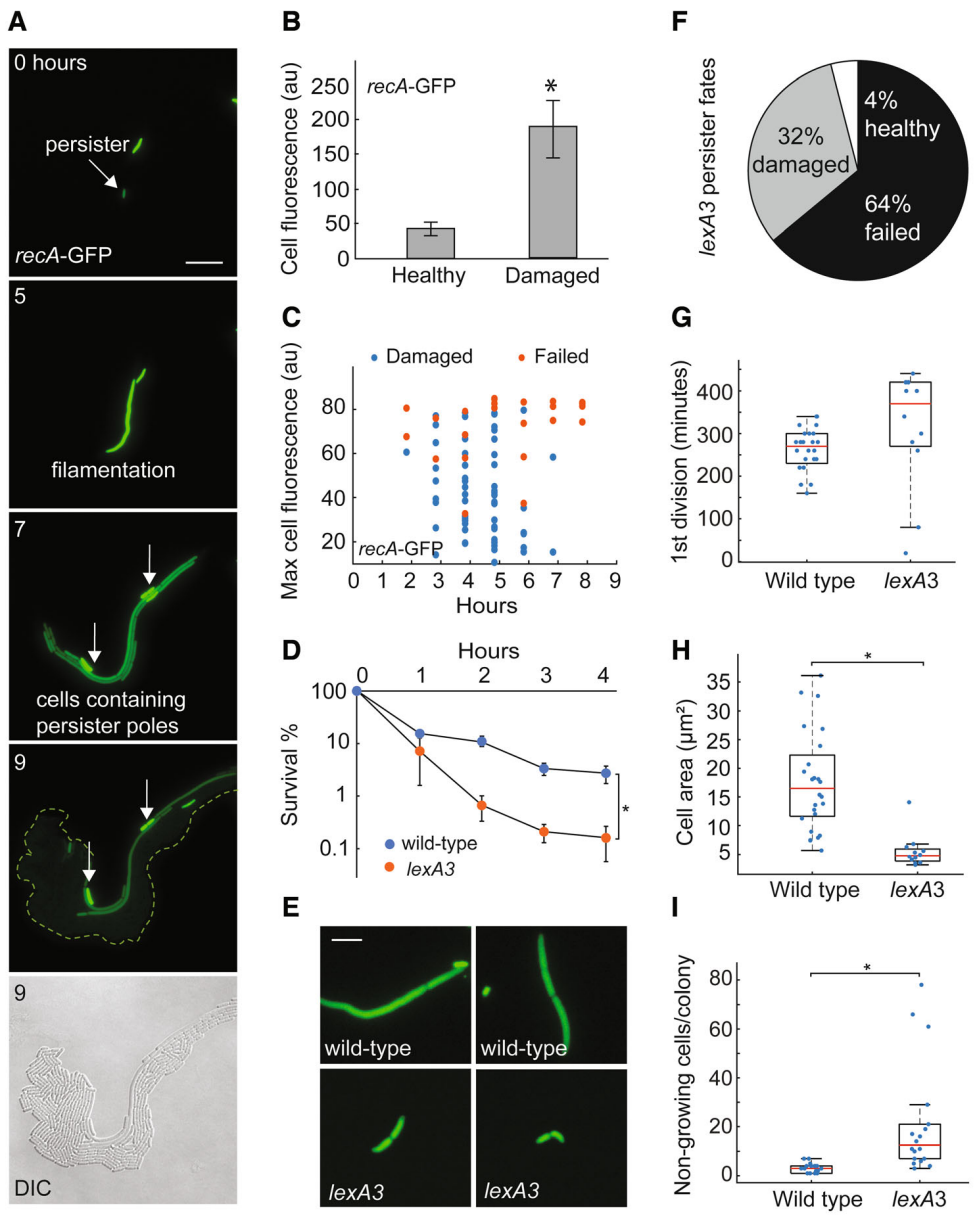
SOS response enables survival and persister partitioning after norfloxacin treatment Representative example of resuscitation in norfloxacin persister carrying fluorescent transcriptional reporter *recA*‐GFP. The same images are used in the Appendix Fig S30. Scale bar: 8 μm.Fluorescence of healthy and damaged persister progeny cells containing *recA*‐GFP 6 h after the end of norfloxacin treatment (values represent mean ± SD, *n* = 27; * indicates *P* < 0.01 by Student's *t* test).Maximum cell fluorescence in individual damaged or failed persisters containing *recA*‐GFP after norfloxacin treatment. Fluorescence values are plotted against resuscitation time (for damaged persisters, *n* = 63) or evident death time (for failed persisters, *n* = 23).Survival curve wide‐type and *lexA3* cells treated with 5 μg/ml norfloxacin (values represent mean ± SD, *n* = 3, biological replicates; * indicates *P* < 0.01 by student's *t* test).Representative examples of 1^st^ division in wild‐type and *lexA3* persisters after norfloxacin treatment. Scale bar: 3 μm.Pie chart depicting the relative proportions of healthy, damaged, and failed *lexA3* persisters after norfloxacin treatment. Results calculated from three data sets.Time of 1^st^ division in damaged wild‐type persisters (*n* = 24) and *lexA3* persisters (*n* = 12) after norfloxacin treatment (values represent mean ± SD). Tops and bottoms of boxes in box‐whisker plots correspond to the 25^th^ and 75^th^ percentiles, central lines correspond to medians, whisker extents correspond to 1.5× the interquartile range.Cell area at 1^st^ division for damaged wild‐type persisters (*n* = 24) and *lexA3* persisters (*n* = 12) during resuscitation after norfloxacin treatment (values represent mean ± SD, * indicates *P* < 0.01 by student's *t* test). Tops and bottoms of boxes in box‐whisker plots correspond to the 25^th^ and 75^th^ percentiles, central lines correspond to medians, whisker extents correspond to 1.5× the interquartile range.Non‐growing progeny cells within microcolonies derived from individual damaged wide‐type persisters (*n* = 19) and *lexA3* persisters (*n* = 19) after norfloxacin treatment (values represent mean ± SD, * indicates *P* < 0.01 by Student's *t* test). Tops and bottoms of boxes in box‐whisker plots correspond to the 25^th^ and 75^th^ percentiles, central lines correspond to medians, whisker extents correspond to 1.5× the interquartile range.

We next tracked resuscitation of cells containing a fluorescent transcriptional reporter for *recA*, the SOS response master regulator induced by norfloxacin treatment (Appendix Fig S29). Quinolone persisters activated *recA* transcription during resuscitation (i.e., after treatment but before the first cell division) (Fig 7A and B, and Appendix Fig S30, Dataset EV1: Videos D1–D3). After the first division, healthy cells lost fluorescence suggesting that SOS response was active specifically during resuscitation. This fluorescence drop in healthy cells highlighted the nonviable persister daughter cells, which remained bright and nondividing after division and were primarily derived from the poles of the original filamented persister cell (Fig 7A and Appendix Fig S30). We did not note clear differences in growth properties or *recA* expression between damaged and failed persisters (Fig 7C and Appendix Fig S31), which led us to hypothesize that persister partitioning itself might enable survival. To test this, we tracked resuscitation in a *lexA3* strain which cannot activate the SOS response in response to DNA damage (Dataset EV1: Videos E1–E4). As expected, the *lexA3* strain had lower persistence (Fig 7D) and did not filament (Fig 7E), indicating SOS response was required for the uneven cellular partitioning observed during wild‐type resuscitation. The *lexA3* mutation substantially shifted the cell‐fate distribution toward more failed and damaged persisters (Fig 7F and Appendix Fig S32), suggesting SOS‐responses' critical role is during resuscitation, though it may also play a role during treatment. Moreover, although time of first division was similar in wild‐type and *lexA3* persisters (Fig 7G), the later were smaller at division (Fig 7H) and contained many more nonviable cells per microcolony (Fig 7I). These findings demonstrate the SOS‐response is required to survive resuscitation after quinolone treatment and support the idea that persister filamentation and partitioning enable the survival.

### Persister partitioning in the standard persister assay

**Figure 8:**
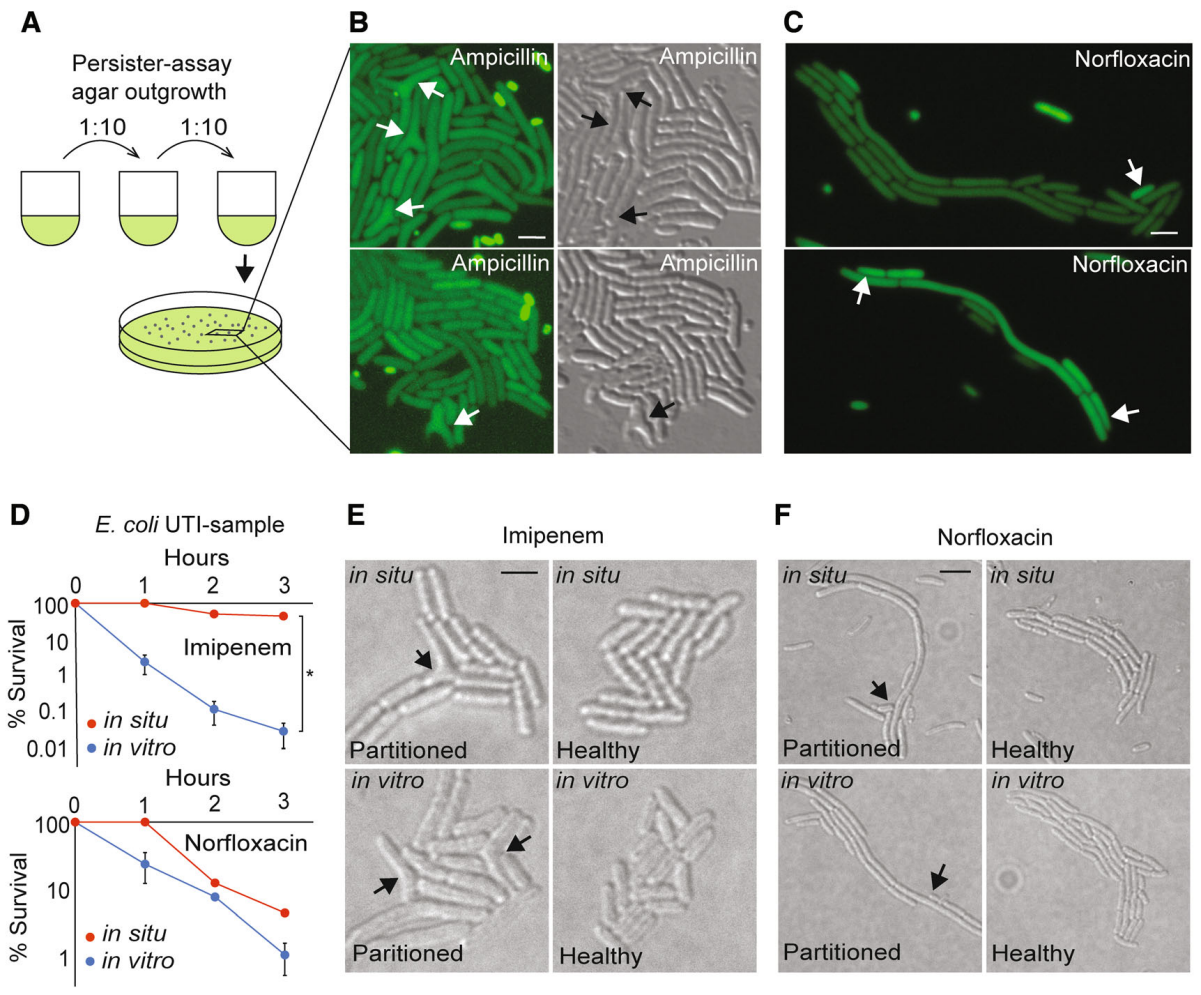
Persister partitioning in the standard persister assay and after *in situ* UTI treatment Diagram illustrating agar outgrowth in the standard persister assay.Representative examples of early microcolonies derived from single ampicillin persisters following standard persister assay with LB agar plating. Cells contained constitutive *ompC*‐GFP for visualization. Arrows indicate damaged cells. Left: fluorescence, right: DIC. Scale bar: 3 μm.Representative examples of early microcolonies derived from single norfloxacin persisters following standard persister assay with LB agar plating. Cells contained constitutive *ompC*‐GFP for visualization. Arrows indicate non‐growing progeny cells. Left: fluorescence, right: DIC. Scale bar: 3 μm.Survival of *in situ*‐treated UTI sample (red) and *in vitro*‐treatment (blue) of the same strains with imipenem or norfloxacin (values represent mean ± SD; *in vitro*: *n* = 3, biological replicates, *in situ*: *n* = 1; * indicates *P* < 0.01 by *t* test).Representative examples of progeny from partitioned (left) and healthy (right) persisters following *in situ* (upper) and *in vitro* (lower) imipenem treatment of an *E. coli* UTI sample. Arrows indicate damaged cells. Scale bar: 3 μm.Representative examples of progeny from partitioned (left) and healthy (right) persisters following *in situ* (upper) and *in vitro* (lower) norfloxacin treatment of an *E. coli* UTI sample. Arrows indicate non‐growing progeny cells. Scale bar: 6 μm.

We speculated persister partitioning might occur in the standard persister assay. Hence, we treated wild‐type *E. coli* with either β‐lactam or quinolone antibiotics, serial diluted cells, and plated on nutrient agar. After allowing time for resuscitation and outgrowth, we excised portions of agar, converted to microscope slides, and imaged (see Materials and Methods). In all cases, we observed identical structural defects, or their lack, as observed using agarose pad methods (Fig 8A–C), indicating persister partitioning is a general aspect of persistence in lab‐strain *E. coli*.

### Persister partitioning in a clinical isolate and an *in situ* UTI

We considered the possibility that persister partitioning might be limited to lab strains of *E. coli*. Hence, we acquired an *E. coli* strain directly isolated from a clinical urinary tract infection (UTI). Urinary tract infections are the most common infections in the United States, and ~80% of them are caused by *E. coli* (Hooton & Stamm, 1997). As before, cells were treated with high doses of ampicillin, imipenem, or ciprofloxacin. Survival dynamics indicated rapid initial death followed by a persistent plateau in cell viability (Appendix Fig S33). For all three antibiotics, we observed cell‐fate trajectories and persister partitioning consistent with our findings in the lab strain, indicating persister partitioning can also occur in clinical strains of *E. coli*.

We sought further evidence of persister partitioning in infections. The majority of *E. coli* in UTIs are present extracellularly, that is, in the urine. Clinicians directly collect samples of this urine to cultivate and test pathogenic microbes. Most of this sample is saved as collected, meaning the bacteria and their environment are unchanged from the *in vivo* infection. We received such a sample from a patient presenting symptoms of a UTI, split it into three volumes and treated *in situ* with imipenem, norfloxacin, or no antibiotic and tracked cell death (Fig 8D). Samples were then washed and placed on agarose pads to observe the resulting resuscitation phenomenon by microscopy. After treatment, we observed antibiotic‐specific persister partitioning consistent with our findings in lab strains (Fig 8E and F, top; Dataset EV1: Videos F1–F4 and G1–G4). We cultured this strain in rich media and performed replicate persister experiments to characterize this strain's persistence and resuscitation behavior. Consistent persister partitioning was observed in these experiments as well (Fig 8E and F, Bottom). *In situ* and *in vitro* quinolone persistence was comparable (Fig 8D); however, *in situ* imipenem persistence of this strain was substantially higher than *in vitro* persistence (Fig 8D). These findings demonstrate that *E. coli* persister partitioning occurs in an *in situ* UTI.

### Persister partitioning in gram‐negative bacteria

To assess the generality of the uncovered persister partitioning, we tracked resuscitation after imipenem or ciprofloxacin treatment in the following gram‐negative pathogenic species: *S. enterica*, *K. pneumoniae*, and *P. aeruginosa*. We observed triangular structural defects and partitioning in all species after imipenem treatment (Appendix Fig S34 and Dataset EV1: Videos H1–H5) and filamentation and polar partitioning in all species after ciprofloxacin treatment. These findings demonstrate that persister partitioning after antibiotic treatment is a general phenomenon.

## Discussion

In this study, we analyzed persister resuscitation from the “bottom up,” that is, combining single‐cell observations and mathematical modeling. Direct observation of resuscitation times provided evidence absent in colony‐appearance‐time data and revealed phenomena masked in bulk populations. We demonstrated that persister resuscitation occurs exponentially after ampicillin treatment in both *E. coli* and *S. enterica*. These dynamics differ from the stochastic model (Balaban *et al*, 2004; Kussell *et al*, 2005; Kaplan *et al*, 2021), which fits the lag‐phase after nutrient limitation and the decay of persister viability during treatment. We found that the model parameters of exponential resuscitation were dependent on antibiotic concentration during treatment and the efflux ability afterward, similar to recent findings on the postantibiotic effect of ribosome targeting antibiotics (Srimani *et al*, 2017). Antibiotic damage was noted in a portion of persisters as they resuscitated. Healthy‐appearing persisters either lack damage or, alternatively, lack sufficient damage to produce visible defects. We currently favor the later explanation as resuscitation times fit a continuous antibiotic‐dependent distribution, perhaps reflecting a continuous spectrum of damage in the persister population. This would suggest that resuscitation times correspond to damage, which could, in turn, be determined by a stochastic exit from lag phase (Balaban *et al*, 2004; Kussell *et al*, 2005; Kaplan *et al*, 2021) during treatment. If true, this would unify the stochastic and exponential models. Contextualized by past research, our findings suggest some persisters are delayed in an antibiotic‐dependent limbo before they resuscitate. This idea is supported by our resuscitation data from quinolone persisters, which are delayed relative to ampicillin persisters and have antibiotic‐specific structural defects and transcriptional responses. This delayed‐limbo hypothesis is also consistent with past studies in *E. coli* demonstrating that some metabolites sensitize persisters to aminoglycosides prior to resuscitation (Allison *et al*, 2011b; Rosenberg *et al*, 2020).

We found persisters follow distinct cell‐fate trajectories during resuscitation, which we currently label healthy, damaged, and failed. Damaged persisters divide to produce both viable and nonviable daughter cells, the latter containing parental cell defects (cell‐wall targeting antibiotics) or parental cell poles (quinolone antibiotics). Similar uneven partitioning was recently observed in a nonpersister study investigating the cellular response to DNA damaging agents (Raghunathan *et al*, 2020). We found failed persisters did not complete division or generate colonies but were distinct from dead (nonviable) cells which did now grow at all and made up majority of the population. Initially, damaged and failed persisters were indistinguishable, having the same cell size and growth characteristics. The only factor we have found so far to differentiate these two cell types is their ability to partition into healthy and unhealthy daughter cells. Our data, particularly with the *lexA3* strain, suggest this partitioning can enable survival, similar to self‐amputation in animals and decay compartmentalization in trees (Shigo & Marx, 1977; Fleming *et al*, 2007), and warrants further investigation. Additionally, as the SOS response induces mutagenic polymerases (Sutton *et al*, 2000), its activation during quinolone persister resuscitation could provide a mechanism for recent reports that persistence facilitates antibiotic resistance (Levin‐Reisman *et al*, 2017; Barrett *et al*, 2019; Windels *et al*, 2019). If correct, quinolone treatment would cause mutation rather than simply select for it.

The persister partitioning we uncovered may provide a survival strategy for bacteria that lack genetic resistance: Rather than evading or inactivating antibiotics, persisters can address treatment in its aftermath by discarding damaged components. This phenomenon was demonstrated in several species (*E. coli*, *S. enterica*, *K. pneumoniae*, and *P. aeruginosa*) with different tolerance and persistence mechanisms, indicating it is common even if persister formation pathways are not. We also demonstrated persister partitioning occurs in the standard persister assay with *E. coli* and should be taken into account in future studies. Persisters are thought to cause biofilm tolerance (Lewis, 2001), though a recent report of a multicellular life cycle in *E. coli* (Puri *et al*, 2023) may suggest that biofilms and persisters form by separate processes. Persisters are also thought to underlie chronic infections (Van den Bergh *et al*, 2017; Moldoveanu *et al*, 2021), though direct evidence connecting laboratory persistence to infections is limited (Balaban *et al*, 2013). Our *in situ*‐UTI evidence support the possibility that persister partitioning happens in patients. These findings could also suggest that *E. coli* persisters, as defined and studied in laboratories (Balaban *et al*, 2019), exist in UTIs. In the future, such *in situ* experiments could be used to firmly establish the proposed roles for persisters in infections.

## Materials and Methods

### Strains

All bacterial strains and plasmids used in this work are listed in Appendix Table S1. *Escherichia coli* K12 MG1655 was used in all experiments unless otherwise indicated. The *hipA7* strain is Th1604 (Korch *et al*, 2003), in which the *hipA7* locus was introduced into MG1655 by Korch *et al* For fluorescence microscopy, a low‐copy plasmid containing pSC101 origin and constitutively expressing GFP*mut2* under the control of *ompC* promoter was transformed into the strains of interest (Zaslaver *et al*, 2006). In our hands, this plasmid produces high and consistent fluorescence across different growth conditions and making it useful for cell tracking. The kanamycin resistance cassette in BW25113 Δ*tolC* strain from the Keio collection was removed by pCP20 thermosensitive plasmid encoding the FLP recombinase as described by Datsenko and Wanner (2000). Wild‐type BW25113 was always used as a control for BW25113 Δ*tolC* experiments. The *E. coli UTI* clinical isolate and UTI sample were provided by Sarah Satola from the Emory University Investigational Clinical Microbiology Core. The *P. aeruginosa* strain was provided by Daniel Wozniak and *S. enterica Salmonella enterica* serovar *typhimurium* LT2 (ATCC 700720) and *Klebsiella pneumoniae* subspecies *pneumoniae* (Schroeter) Trevisan (ATCC 43816) were purchased from American Type Culture Collection.

### Chemicals and antibiotics

Luria‐Bertani Broth, Miller medium (LB, EMD Millipore) was used for bacterial culture in all experiments. Ampicillin (Gold Biotechnology) was used at a concentration of 100 μg/ml unless otherwise stated. Norfloxacin (Sigma) was used at 5 μg/ml for *E. coli* and *P. aeruginosa*. Imipenem (Gold Biotechnology) was used at a concentration of 20 μg/ml for *E. coli*, *S. enterica*, *K. pneumoniae* and clinical UTI isolate, 10 μg/ml for clinical UTI urine sample, and 100 μg/ml for *P. aeruginosa*. 5 μg/ml ciprofloxacin was used for *S. enterica*, *K. pneumoniae*, clinical UTI isolate, and clinical UTI urine sample. The working concentrations of Phenylalanine‐arginine β‐naphthylamide (PAβN, Sigma) were chosen based on previous work to inhibit efflux activity while minimally affecting growth (Bohnert *et al*, 2010; Matsumoto *et al*, 2011). 25 μg/ml kanamycin (Gold Biotechnology) was included in all cultures where cells contained reporter plasmids (p*ompC::gfpmut2*, p*pbpG::gfpmut2* or p*recA::gfpmut2*). Phosphate‐buffered saline pH 7.2, (PBS, BD) was used to wash cells.

### Bacterial growth, treatment, and persister resuscitation conditions

For persister assays, exponential phase cultures of bacteria were inoculated 1:1,000 into fresh LB and grown at 37°C in a shaking incubator at 300 rpm for 16 h. Cultures were supplemented with kanamycin (25 μg/ml) when required for plasmid retention. Stationary phase culture was then diluted 1:50 or 1:33 into fresh LB with ampicillin (100 μg/ml) or other antibiotics at concentrations specified above and incubated at 37°C and 300 rpm. Colony‐forming units taken at regular intervals indicated killing plateaued by 3 h (Appendix Fig S1). For resuscitation experiments, cultures were centrifuged after 3 h of treatment and washed with PBS and LB to remove antibiotics. Cells were then re‐suspended in fresh LB, and added onto 2% agarose/LB pads on microscope slides (VWR; Young *et al*, 2011). Agarose pads were then incubated in a 37°C incubator until imaging by microscopy, or in the case of cell tracking, experiments were incubated in a stage‐top‐incubator on the microscope. Resuscitation of clinical UTI strain was performed similarly except for treating with antibiotics for 3.5 h. For efflux experiments, PAβN was included in both washing media and agarose pad to ensure uniform concentration but was not included during antibiotic treatment. To investigate the effect of cell density on resuscitation, stationary phase MG1655 cells carrying p*ompC::gfpmut2* (green) or pEB2‐mScarlet (red) plasmids were separately treated with ampicillin as described above. Cells were then washed with PBS and resuspended in LB with ratios of red/green cells: 1:1, 2:1, 4:1, 8:1, 16:1, and 32:1. The mixed cells were loaded on to different LB agarose pads for incubation at 37°C and imaged by microscope to track resuscitation.

### Microscopy

All images were collected using a Zeiss Apotome.2 microscope, using a Plan‐Apochromat 63×/1.4 NA DIC objective and an Axiocam 503 camera or a Leica DMi8 microscope equipped with a DIC HCPL APO 63× oil immersion objective (1.6× mag changer) Hamamatsu ORCA‐Flash 4.0 camera, and Lumencor Spectra‐X light engine. At indicated time points, tiled images containing 24–42 frames were acquired at several slide locations. For time‐series experiments on the Zeiss microscope (Fig 1), in which images of the same cells were captured every 30 or 60 min, the *x*, *y*, and *z* coordinates of the initial slide position were recorded in order to locate the precise position at subsequent time points. Sample slides, made simultaneously with experimental slides, were used to initialize the focal plane and stage angle of the microscope prior to each experiment. For time serious experiments on the Leica, including the cell partitioning and cell‐fate experiments, a stage‐top incubator (Tokai‐Hit, STX Stage Top Incubator Temp and Flow; STXF‐WSKMX‐SET), was used and both the incubator and the microscope objective were maintained at 37°C prior to and throughout all experiments. In these cases, Leica LAS X software was programmed to automatedly acquire tiled images (25–80 fields of view per image) at regular and specific time intervals at a 100×. Images were acquired in the differential interference contrast (DIC) configuration or in the red (excitation 510 nm; emission 592–668 nm) or green (excitation 470 nm; emission 500–550 nm) fluorescent channels.

### Image analysis

For bright‐field micrographs, cells per microcolony were enumerated in ImageJ. For fluorescent micrographs used to study dynamics, a custom Matlab script identified cells and clustered them into microcolonies. Briefly, raw images were re‐scaled, edge detection was employed using a Laplacian of Gaussian filter, and object area and centroid were calculated. Broad volume and width constraints were imposed on cells; shape constraints were not employed to avoid any biasing. Accuracy was confirmed by mapping centroids onto original microscopy images (see Appendix Fig S5 for example). Microcolony centroids were identified after filling and dilating the cell edges, and cells were clustered by colony centroids to determine the number of cells in each microcolony. ImageJ was used to quantify the fluorescent intensity in GFP‐transcriptional reporter experiments (*pbpG* and *recA*) in persisters and their progeny.

### Data analysis

All data were analyzed in Matlab. All figures were generated using Matlab or Excel and formatted with Adobe Illustrator. Values for parameters and variables were determined as described below. For bright‐field microscopy data, the resuscitation time (*t*_*R*_) was defined as 30 min prior to the first observation of two cells in a microcolony. In the rare cases in which a cell appeared to go from 1 to 3 cells, *t*_*R*_ was defined as 45 min prior. For fluorescence microscopy, the *t*_*R*_ was calculated from the number of cells per colony (Appendix Fig S4) using the following equation:

$$t_{R}=t-\mu \;\log_{2}\left(\frac{\text{cells}}{\text{colony}}\right).$$

The normalized cell resuscitation during discrete time interval (*ΔP*_*t*_) was calculated from the t_R_ values and normalized by the total number of observed resuscitation events. For bright‐field microscopy data, the cellular division rate (*μ*) was determined by calculating the logarithm of the difference in cells per colony at two‐cell state and 1 h (*Δt*) after the two‐cell state using the equation below:

$$\mu =\frac{\log_{2}\left({\text{cells}}_{2}-{\text{cells}}_{1}\right)}{\mathrm{\Delta t}}$$

Values for *k* were calculated from the model equation by dividing the *ΔP*_*t*_ by the number of cells remaining in persistent state over a discrete time interval. Given the apparent linearity of ln (*k*), an exponential curve was fit to *k* and *R* ^*2*^ was calculated. The normalized number of cells in the persistent state that have not resuscitated at a particular time (*P*_*t*_), was determined using the following equation (*P*_*0*_ is the total number of cells that resuscitated):

$$P_{t}=\frac{P_{0}-{\mathrm{\Delta P}}_{t}}{P_{0}}.$$

The time‐dependent exponential relationship for *k* was substituted into the model equation (equation 1) yielding equation (3) which was then integrated to derive an equation for resuscitation (equation 4). Equations for the exponential and stochastic models were fit to observed data, and parameters were estimated using Matlab. Models were compared by plotting residuals and using the *χ* ^2^ method for nested models.

### Stationary phase resuscitation

To assess the effect of the Δ*tolC* mutation and PAβN treatment on the resuscitation of cells after nutrient limited stationary phase, cultures were grown in LB for 16 h at 37°C. Cells were then diluted 1:100 into fresh LB without antibiotic and were directly loaded onto LB agarose pad and incubated at 37°C. For the PAβN case, 100 μg/ml PAβN was added to fresh LB agarose pad.

### Tracking resuscitation in a standard persister assay

Stationary phase MG1655 cells carrying p*ompC::gfpmut2* were diluted into fresh LB and treated with 100 μg/ml ampicillin or 5 μg/ml norfloxacin for 3 h. Cultures were then centrifuged at 8,000 *g* for 2 min to collect cells, which were then washed once in PBS, then serially diluted in PBS and spot plated on LB agar plates. Plates were incubated at 37°C incubator for 3–5 h. After incubation, ~25 × 75 mm slices of LB agar cut from plates and moved onto glass microscope slides. The agar slide containing cells was gently covered with cover slips and slides were imaged by microscopy.

### Resuscitation after *in situ* UTI treatment

A patient's urine sample was received from the Emergency Department at Emory University hospital. The patient was an 88‐year‐old woman with a suspected UTI. She did not have an indwelling catheter and was not taking antibiotics at the time. The Emory Clinical Microbiology Laboratory identified *E. coli* with broad antibiotic susceptibly at 10^5^ CFU/ml in the sample. The urine sample was stored at 4°C before experiments. Prior to experiments, the sample (~5 ml) was split into three 1‐ml volumes and incubated in 15‐ml culture tubes at 37°C. Tubes were then treated with 10 μg/ml imipenem, 5 μg/ml norfloxacin, or no treatment and aliquots were taken every hour and serial diluted to determine viability. After 3 h of treatment, samples were centrifuged at 8,000 *g* for 2 min and washed with PBS. Cells were then resuspended in fresh LB and added to LB agarose pads. Resuscitation was tracked by microscopy as described above. As a control, we streaked the urine sample on the LB agar and randomly picked individual colonies which were then studied for persister resuscitation as described above.

## Author contributions

**Xin Fang:** Data curation; formal analysis; validation; investigation; visualization; methodology; writing – original draft. **Kyle R Allison:** Formal analysis; supervision; funding acquisition; investigation; writing – original draft; writing – review and editing.

## Disclosure and competing interests statement

The authors declare that they have no conflict of interest.

## Supporting information



Appendix

Dataset EV1

## Data Availability

Data supporting the findings of this study are available in the Appendix.
