# Resuscitation dynamics reveal persister partitioning after antibiotic treatment

**DOI:** 10.15252/msb.202311672

**Published:** 2023-05-04

**Authors:** Xin Fang, Kyle R Allison

## Abstract

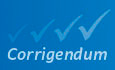

**Correction to**: *Mol Sys Biol* (2023) 4: e11320. DOI: 10.15252/msb.202211320 | Published online 3 March 2023

**Sentences are added to the Discussion.**

**Three citations are added to the Discussion section of this paper.**

The following sentences are added to the Discussion (Text in bold):

“If true, this would unify the stochastic and exponential models. **Separately, recent studies have applied the term “persister” to growth‐arrested cells, achieved by rifampicin treatment (Kim *et al*, 2018; Yamasaki *et al*, 2020). Nearly all of these cells then survive ampicillin treatment and have heterogeneous fates including some which return to exponential growth. This “tolerance” is expected given that ampicillin killing is strictly proportional to cellular growth rate (Tuomanen *et al*, 1986). Moreover, the resuscitation of these cells likely reflects the recovery from rifampicin‐induced ribosome depletion (Hamouche *et al*, 2021).** Contextualized by past research, our findings suggest some persisters are delayed in an antibiotic‐dependent limbo before they resuscitate.”

The following three citations are added to the Discussion section of this paper.

Kim J‐S, Yamasaki R, Song S, Zhang W, Wood TK (2018) Single cell observations show persister cells wake based on ribosome content. *Environ Microbiol* 20: 2085–2098.

Tuomanen E, Cozens R, Tosch W, Zak O, Tomasz A (1986) The rate of killing of Escherichia coli by beta‐lactam antibiotics is strictly proportional to the rate of bacterial growth. *J Gen Microbiol* 132: 1297–1304.

Hamouche L, Poljak L, Carpousis AJ (2021) Ribosomal RNA degradation induced by the bacterial RNA polymerase inhibitor rifampicin. *RNA* 27: 946–958.

We apologize for any inconvenience this omission may have caused.

