# Supplementary material for: Resuscitation dynamics reveal persister partitioning after antibiotic treatment
Source: Mol Syst Biol. 2023 Mar 3;19(4):e11320. doi: 10.15252/msb.202211320 (PMC10090945; doi:10.15252/msb.202211320)
Supplement: Supplementary file 1 — Appendix [file MSB-19-e11320-s002.pdf]

Appendix: Resuscitation dynamics reveal persister partitioning after antibiotic treatment

Xin Fang and Kyle R. Allison*

Wallace H. Coulter Department of Biomedical Engineering, Emory University and Georgia Institute of Technology, Atlanta, GA, USA;
Department of Medicine/Division of Infectious Diseases, Emory University School of Medicine, Atlanta, GA, USA

*Corresponding author: Kyle R. Allison; email: kyle.r.allison@emory.edu; phone: (404) 727-6974

Table of Contents

Appendix Table S1 3
Appendix Figure S1 4
Appendix Figure S2 5
Appendix Figure S3 6
Appendix Figure S4 7
Appendix Figure S5 8
Appendix Figure S6 9
Appendix Figure S7 10
Appendix Figure S8 11
Appendix Figure S9 13
Appendix Figure S10 14
Appendix Figure S11 15
Appendix Figure S12 16
Appendix Figure S13 17
Appendix Figure S14 18
Appendix Figure S15 19
Appendix Figure S16 20
Appendix Figure S17 21
Appendix Figure S18 22

Appendix Figure S19 23
Appendix Figure S20 24
Appendix Figure S21 25
Appendix Figure S22 26
Appendix Figure S23 27
Appendix Figure S24 28
Appendix Figure S25 29
Appendix Figure S26 30
Appendix Figure S27 31
Appendix Figure S28 32
Appendix Figure S29 33
Appendix Figure S30 34
Appendix Figure S31 35
Appendix Figure S32 36
Appendix Figure S33 37
Appendix Figure S34 38
Additional References 39

Strains/plasmids	Genotype/description	Source
MG1655	*E. coli* K-12 F- , λ-, *ilvG, rfb-50, rph-1*	ATCC
hipA7	MG1655 *hipA7 zde264::Tn10 dapA6*	(Korch *et al*, 2003)
BW25113	*E. coli* K-12 *F-, Δ(araD-araB)567, ΔlacZ4787(::rrnB-3), λ-, rph-1, Δ(rhaD-rhaB)568, hsdR514*	Yale strain stock
tolC	BW25113 *ΔtolC* (Kan cassette removed)	Keio Collection(Baba *et al*, 2006)
tolC	MG1655 *ΔtolC* (Kan cassette removed)	This study
lexA3	*E. coli* K-12 F- , λ-, *ilvG, rfb-50, rph-1 lexA3*(Ind-)	This study
pUA66 *pompC::gfp*	pUA66 derivative containing the *gfpmut2* gene under control of the *ompC* promoter	(Zaslaver *et al*, 2006)
pUA66 *ppbpG::gfp*	pUA66 derivative containing the *gfpmut2* gene under control of the *pbpG* promoter	(Zaslaver *et al.*, 2006)
pUA66 *precA::gfp*	pUA66 derivative containing the *gfpmut2* gene under control of the *recA* promoter	(Zaslaver *et al.*, 2006)
pEB2-mScarlet-I	pEB2 derivative containing the mScarlet-I coding sequence under constitutive promoter	(Balleza *et al*, 2018)
pCP20	Thermosensitive helper plasmid encoding the FLP recombinase	(Datsenko & Wanner, 2000)
Pseudomonas aeruginosa PAO1	*lacIq*+ *Δ*(lacZ)M15+ *tetA*+ *tetR*+ (ATCC 47085)	(Pestrak *et al*, 2018)
S. enterica serovar Typhimurium LT2	ATCC 700720	ATCC
Klebsiella pneumoniae	ATCC 43816	ATCC
UTI *E.coli* strain	Clinical isolate stock from Emory hospital	This study

Appendix Table S1: Bacterial strains and plasmids used in this work

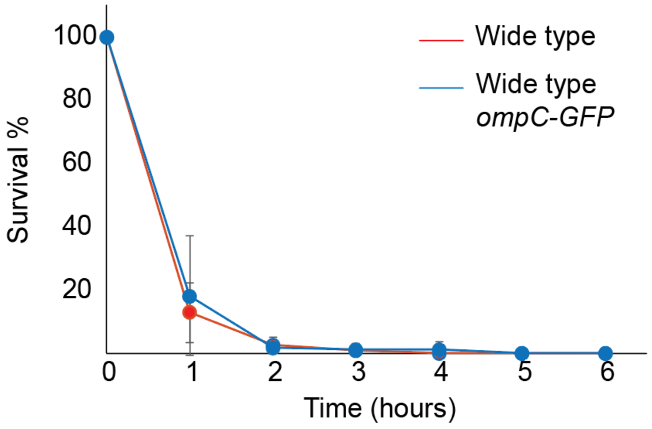

Appendix Figure S1. Survival of MG1655 and MG1655 with pUA66 *pompC::gfp* grown 16 hours in LB then treated in fresh LB with 100 µg/mL ampicillin. At the designated time points, samples were serially diluted and spot plated on LB agar to determine colony-forming units (CFUs). Survival data are plotted as the mean across three biological replicates and error bars indicate standard deviation (SD).

Deleted: experiments

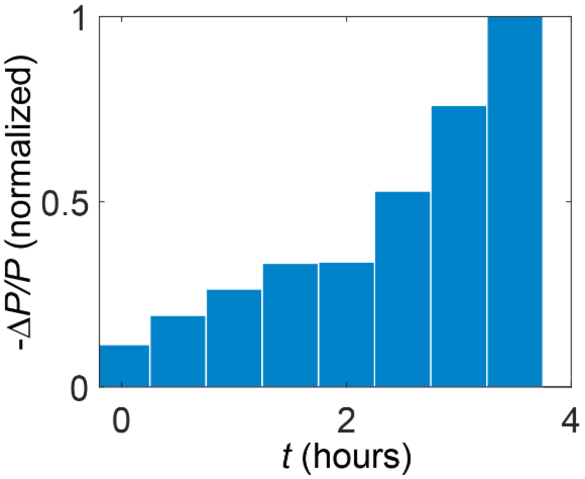

Appendix Figure S2. Fraction of cells leaving persistent state after ampicillin treatment. Histogram of remaining cells transitioning out of the persistent state ($-\Delta P/P$) as a function of time (t) (n = 228 cell lineages, same dataset as Fig. 1C).

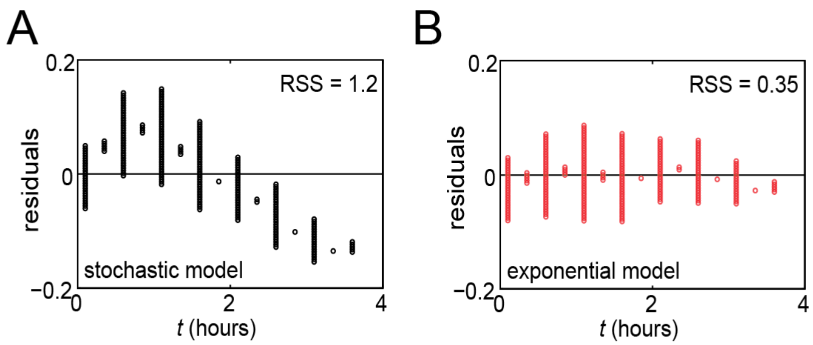

Appendix Figure S3. Residuals analysis for the stochastic **(A)** and exponential **(B)** models versus directly observed resuscitation times**.** The residual sum of squares (RSS) for both models is indicated on plots.

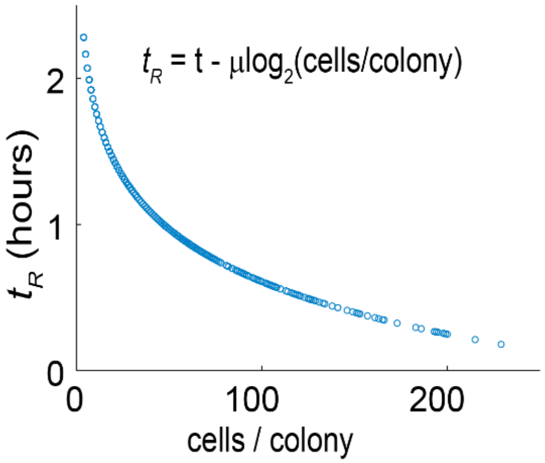

Appendix Figure S4. Imputation of t_R (resuscitation time) from cells per colony. The resuscitation time (t_R) was imputed from the numbers of cell per colony identified computationally and the growth equation displayed in the figure.

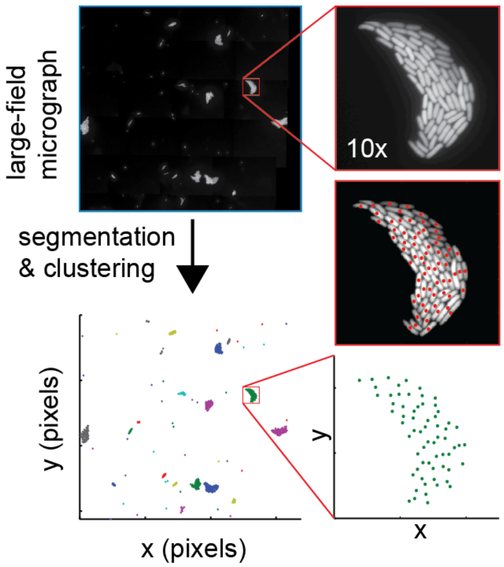

Appendix Figure S5. Computational segmentation and clustering. Illustration of endpoint fluorescence microscopy for determination of resuscitation time of persistent bacteria. After ampicillin treatment, persistent bacteria harboring a constitutively-expressed fluorescent protein were grown at 37°C in the absence of antibiotic on fresh medium and imaged after 3 hours. Tiled images have been acquired and stitched. The Laplacian of Gaussian method was applied to images for edge detection and cell identification, and cells were enumerated and clustered computationally. The same image was used in Figure 2D.

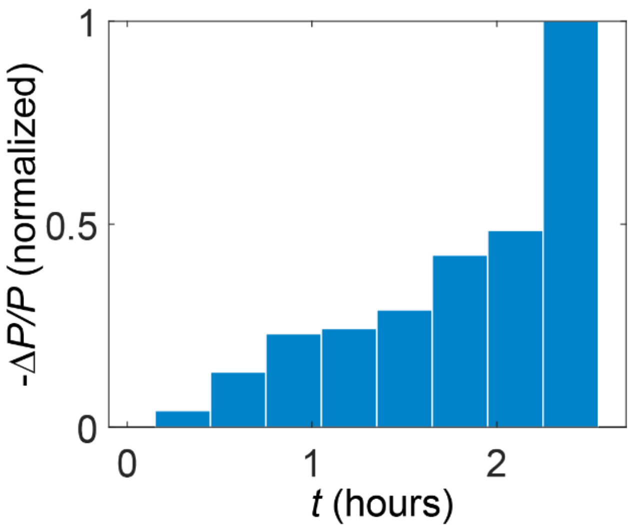

Appendix Figure S6. Fraction of cells leaving persistent state from fluorescence data. Histogram of imputed remaining cells transitioning out of the persistent state ($-\Delta P/P$) as a function of time (*t*) (n = 645 cell lineages).

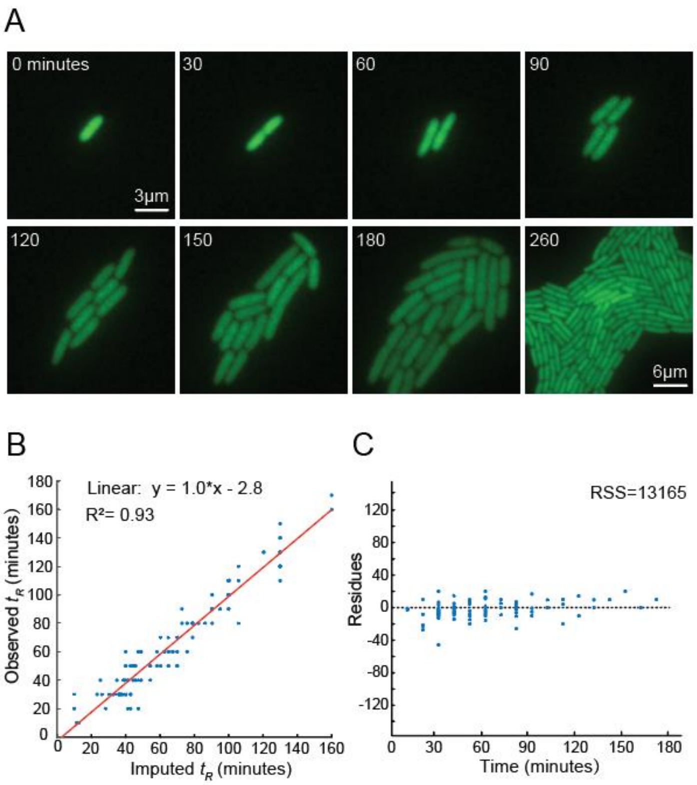

Appendix Figure S7. Imputed resuscitation time (t_R) predicts directly observed t_R values. **(A)** Example micrographs of *E. coli* MG1655 persister carrying pUA66 *pompC::gfp* reporter resuscitation after ampicillin (100 μg/mL) treatment. Microcolony doubling time after resuscitation was ~30 minutes. (**B**) Scatter plots of imputed versus observed t_R. The imputed t_R was calculated from cells per colony and the growth equation displayed in Appendix Fig S4. (**C**) The residuals between imputed and observed t_R.

Deleted: resuscition

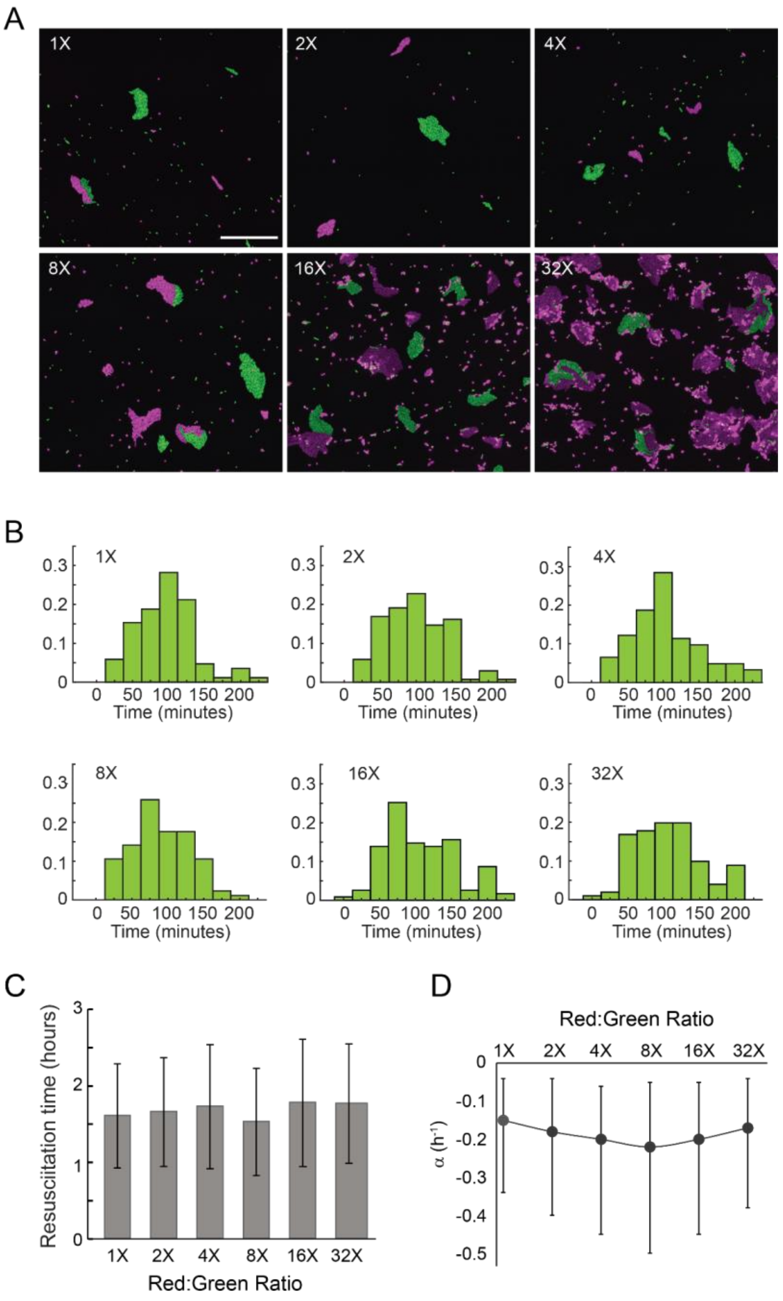

A
1X
2X
4X
8X
16X
32X
B
Time (minutes)
C
Resuscitation time (hours)
Red:Green Ratio
D
Red:Green Ratio
α (h⁻¹)

Appendix Figure S8. Density dependence of resuscitation after ampicillin treatment. **(A)** Representative micrographs of ampicillin persister resuscitation on LB agarose pad with different cell densities. Wild-type ampicillin persisters containing either green (GFP) or red (mScarlet, depicted in magenta) fluorescent proteins were mixed at a range of ratios and allowed to resuscitate. The Red:Green ratio (*e.g.*, "4X") is indicated on each micrograph and the scale bar represents 50 µm. **(B)** Histograms of resuscitation times of GFP labelled persisters, with different Red:Green ratios (1X, n=85; 2X, n=136; 4X, n=123; 8X, n=85; 16X, n=115; 32X, n=101). **(C)** Mean and standard resuscitation times for the distributions in **(B)**. **(D)** α-values computed by fitting the exponential model to the data in **(B)**. Plot represents α ± 95% confidence intervals.

Deleted: by

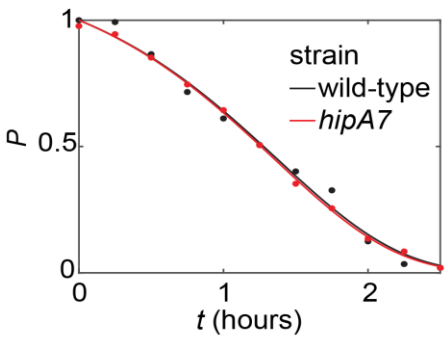

Appendix Figure S9. Persister resuscitation dynamics of wild-type and *HipA7* E. coli after treatment with 100 µg/mL ampicillin. Cells in persistent state (*P*) over time (*t*) for wild-type and *hipA7* strains (data binned into 10 time points; n = 131 and 212 for wild-type and *hipA7*, respectively) with exponential model fit curves.

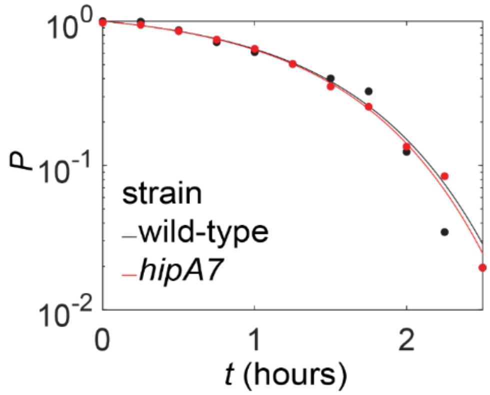

Appendix Figure S10. Persister resuscitation dynamics (log-scale) of wild-type and *HipA7* E. coli after treatment with 100 µg/mL ampicillin. Cells in persistent state (*P*) over time (*t*) for wild-type and *hipA7* strains (data binned into 10 time points; n = 131 and 212 for wild-type and *hipA7*, respectively) with exponential model fit curves.

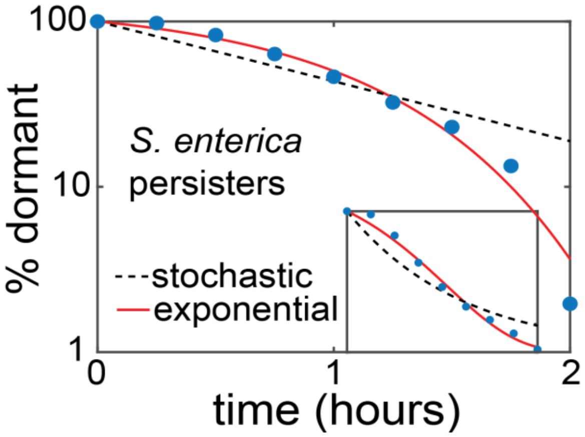

Appendix Figure S11. Persister resuscitation dynamics of *S. enterica* after treatment with 100 µg/mL ampicillin. Cells in persistent state (*P*) over time (*t*) for wild-type *S. enterica* strains with both stochastic and exponential model fit curves. Insert depicts linear-scale of the same data.

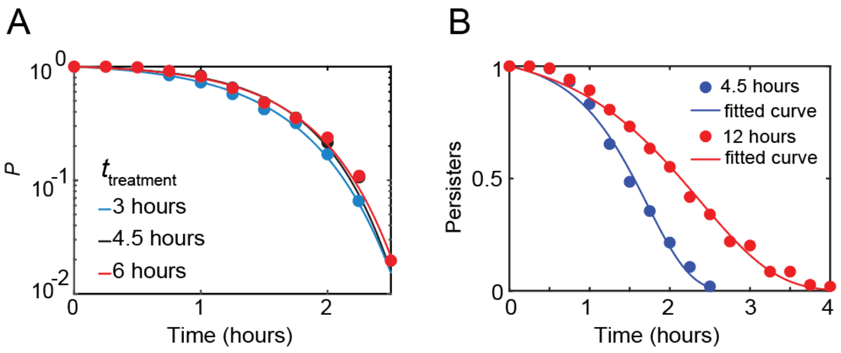

Appendix Figure S12. Persister resuscitation dynamics after treatment with 100 µg/mL ampicillin for varying times. **(A)** Cells in persistent state (*P*) over time (*t*) after initial antibiotic treatment for 3, 4.5, or 6 hours. Data binned into 10 time points with exponential-model fit curves (n = 334, 181, and 153 for 3, 4.5, and 6 hour treatments, respectively). **(B)** Cells in persistent state (*P*) over time (*t*) after initial antibiotic treatment for 4.5 (n=181) or 12 hours (n=431). Exponential model curves are fit to data.

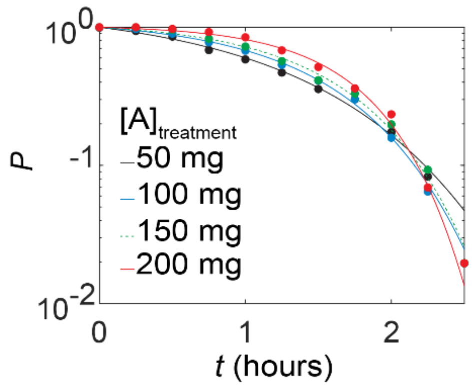

Appendix Figure S13. Persister resuscitation dynamics after treatment with varying concentrations of ampicillin. Cells in persistent state (*P*) over time (*t*) after initial treatment with different ampicillin concentrations ($[A]_{treatment}$) (data were binned into 10 time points; n = 279, 261, 214, and 178 microcolonies for 50, 100, 150, and 200 µg/mL, respectively) with exponential-model fit curves.

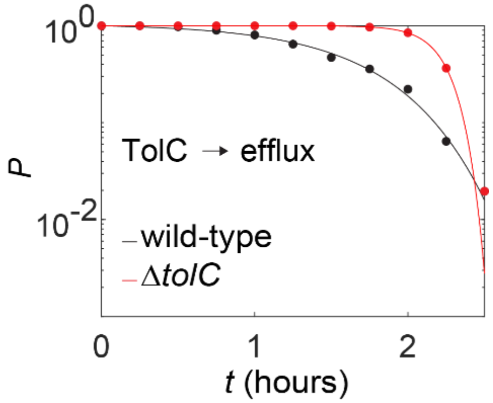

Appendix Figure S14. Role to *tolC* in persister resuscitation dynamics after treatment with 100 μg/mL ampicillin. Cells in persistent state (*P*) over time (*t*) in wild-type (BW25113) and efflux-impaired Δ*tolC* strains (data binned into 10 time points; n = 330 and 122 microcolonies for wild type and Δ*tolC*, respectively) with exponential-model fit curves.

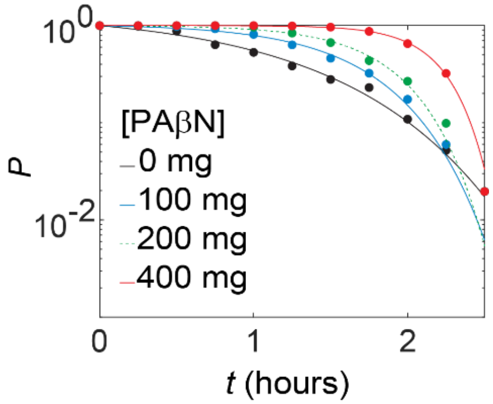

Appendix Figure S15. Inhibiting efflux delays persister resuscitation after ampicillin treatment. Cells in persistent state (*P*) over time (*t*) with differing concentrations of efflux inhibitor phenylalanine-arginine β-naphthylamide ([PAβN]) (data binned into 10 time points; n = 121, 171, 186, and 107 microcolonies for 0, 50, 100, and 200 μg/mL, respectively) with exponential-model fit curves. PAβN was only added during resuscitation and was not present during antibiotic treatment.

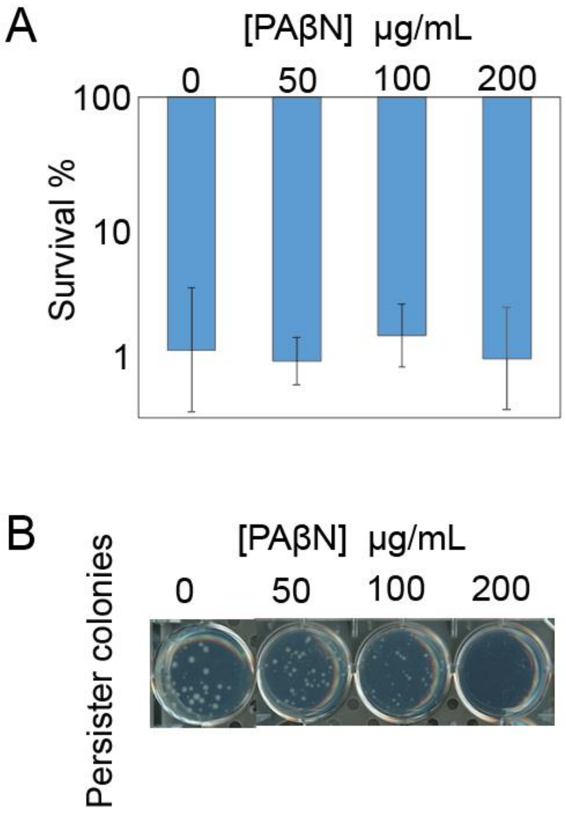

Appendix Figure S16. PAβN does not affect number of resuscitating persisters. **(A)** % survival determined by CFU/mL with PAβN concentrations of 0, 50, 100, and 200 μg/mL in LB agar plates. **(B)** Example on LB agar of colonies formed from persisters after 16 hours growth in the presence of PAβN. Colonies appear later in the presence of PAβN, but equivalent number of colonies are observed.

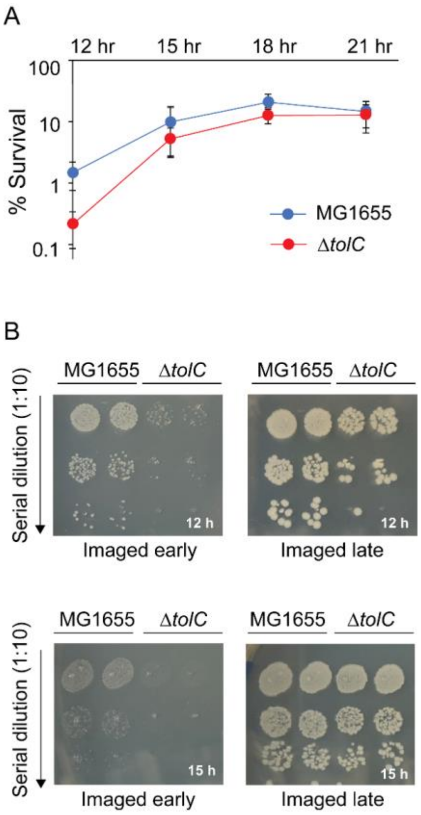

Appendix Figure S17. Effect of Δ*tolC* mutation on persister survival. **(A)** Persistence as a function of growth. Cultures were grown for 12, 15, 18, 21 hours then treated with 100 μg/mL ampicillin for 3 hours to determine survival. **(B)** Representative images showing the delayed growth of Δ*tolC* persister colonies after resuscitation compared to wild-type (MG1655) persisters. Images of persisters derived from 12- and 15-hour overnight culture were taken around 8 hours (early) and 21 hours (late) after plating.

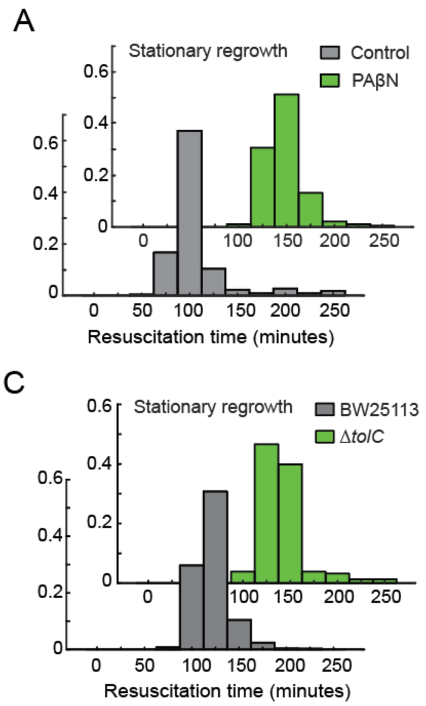

Appendix Figure S18. The effect of efflux activity on stationary-phase *E. coli* regrowth in fresh LB. **(A)** Impact of 100 µg/mL PAβN on lag time distributions of MG1655 cell-regrowth in fresh LB from stationary-phase culture (n=221 and 189 for control and PAβN, respectively). **(B)** Resuscitation curves for stationary phase MG1655 grown with or without 100 µg/mL PAβN in the fresh LB (n=221 and 189 for control and PAβN, respectively). **(C)** Effect of *tolC* mutation on lag time distributions of BW25113 cell-regrowth in fresh LB from stationary-phase culture (n=249 and 311 for wild-type BW25113 and *ΔtolC*, respectively). **(D)** Resuscitation curves for stationary phase wild-type (BW25113; n = 249) and efflux-impaired Δ*tolC* (n = 311) strains in fresh LB.

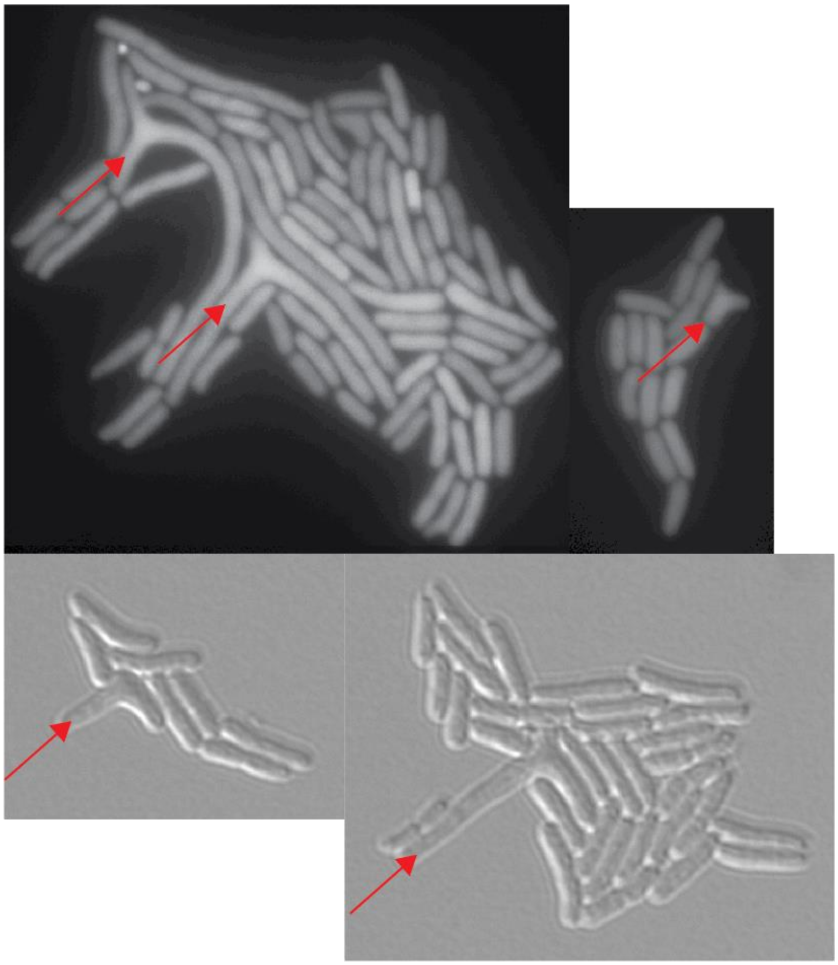

Appendix Figure S19. Antibiotic damage in persister progeny. Example microcolonies deriving from single persister cells after resuscitation from 100 µg/mL ampicillin treatment. Red arrows indicate cells with structural defects consistent with ampicillin mode of action.

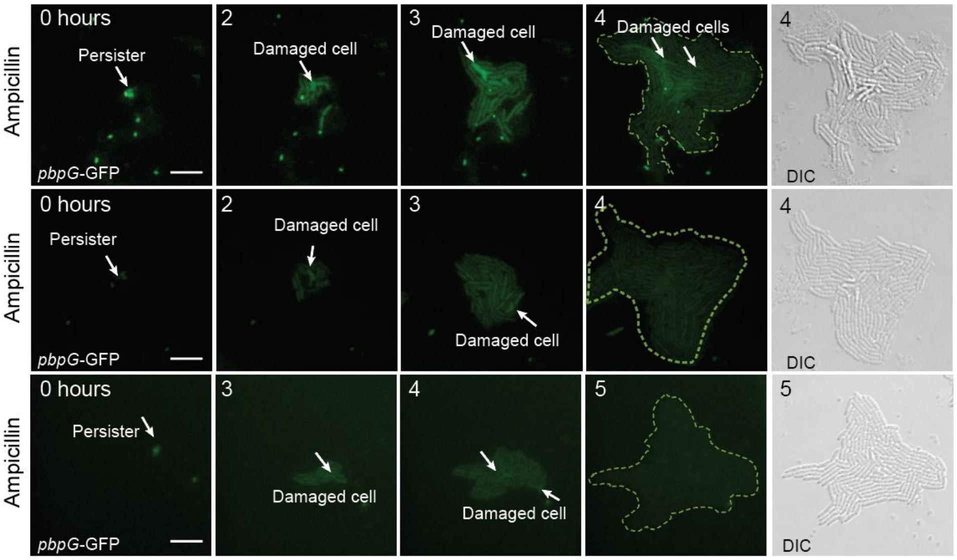

Appendix Figure S20. *pbpG* expression during persister resuscitation from 100 μg/mL ampicillin treatment. Example time courses of resuscitating persisters carrying a *pbpG*-GFP transcriptional reporter (pUA66 *ppbpG::gfp*). Scale and time are indicated on images. Dashed line indicates microcolony boundary. Scale bar: 8 μm.

Deleted: induction

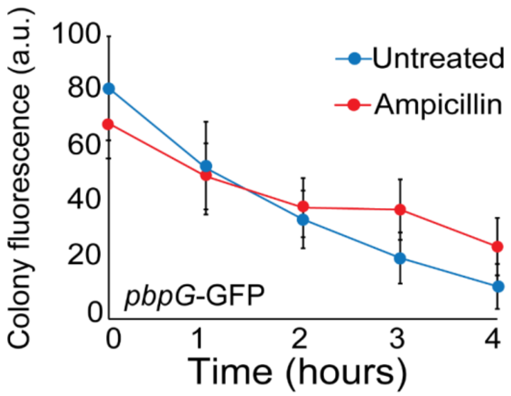

Appendix Figure S21. Cellular fluorescence for resuscitating colonies after treatment with 100 µg/mL ampicillin or no treatment. Transient *pbpG* induction for colonies derived from a single ampicillin persister. Colony *pbpG*-GFP intensity over time for microcolony derived from MG1655 ampicillin persisters or resuscitating untreated stationary phase cells. Values represent mean ± SD, n=6, biological replicates

Deleted: .

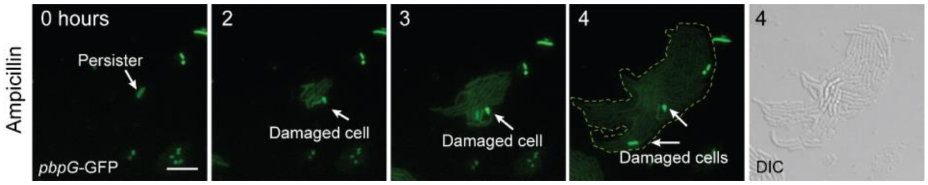

Appendix Figure S22. *pbpG* expression and persister partitioning after 100 µg/mL ampicillin treatment. Example time courses of resuscitating persisters carrying a *pbpG*-GFP transcriptional reporter. Scale and time are indicated on images. Dashed line indicates microcolony boundary. Scale bar: 8 µm.

Deleted: induction

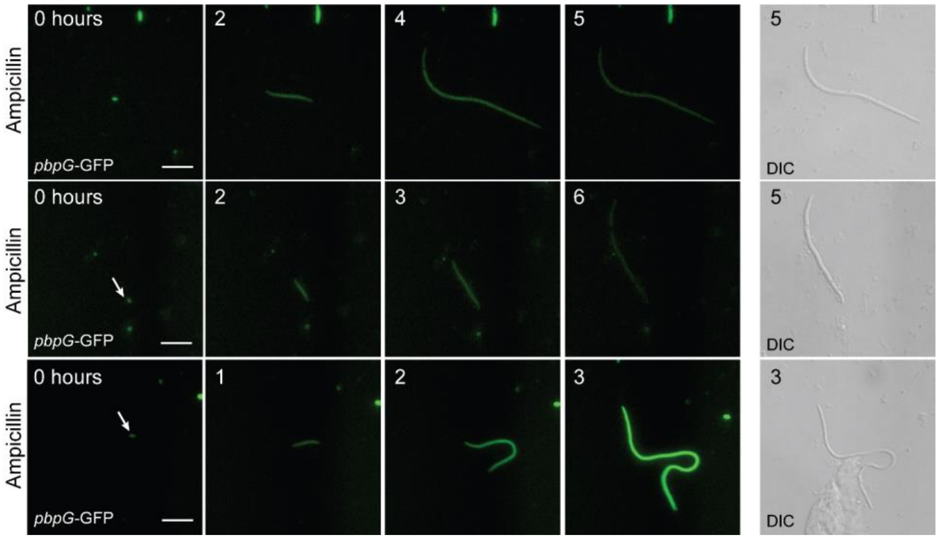

Appendix Figure S23. Representative micrographs of "failed" persisters carrying *pbpG*-GFP reporter after 100 µg/mL ampicillin treatment. Scale and time are indicated on images. Scale bar: 8 µm.

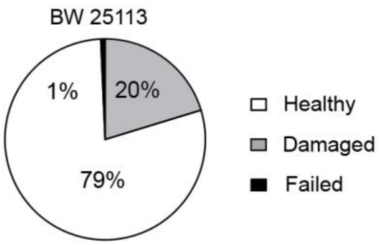

Appendix Figure S24. Pie chart depicting the proportion of resuscitated BW25113 persisters that are healthy, damaged, or fail after 100 µg/mL ampicillin treatment.

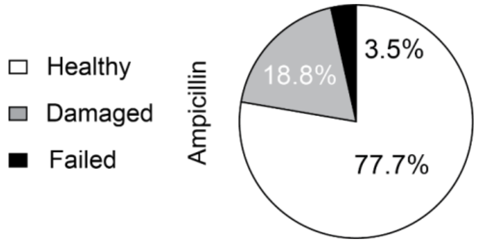

Appendix Figure S25. Pie chart depicting the proportion of resuscitated MG1655 persisters that are healthy, damaged, or fail after 200 µg/mL ampicillin treatment.

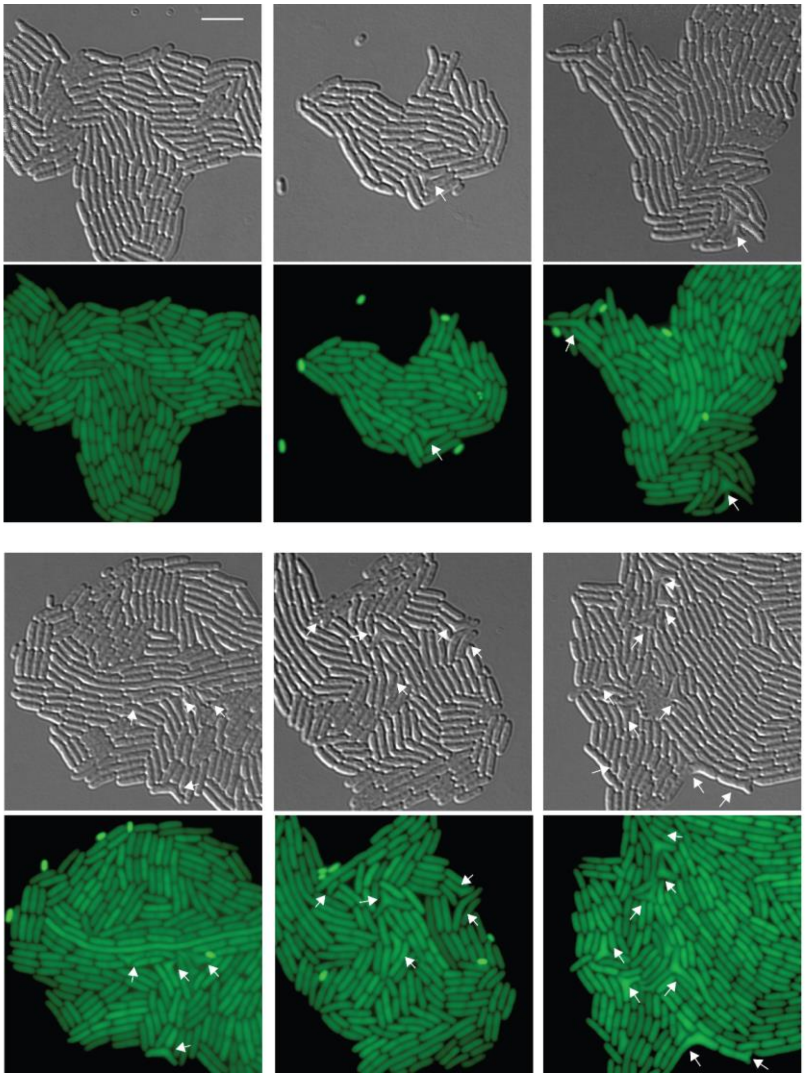

Appendix Figure S26: Representative micrographs of microcolonies deriving from single persister cells taken several hours after resuscitation from 100 µg/mL ampicillin treatment (top: DIC; bottom: fluorescence). Microcolonies can harbor multiple cells with structural defects, indicated with white arrows. Scale is indicated on images. Scale bar: 6 µm.

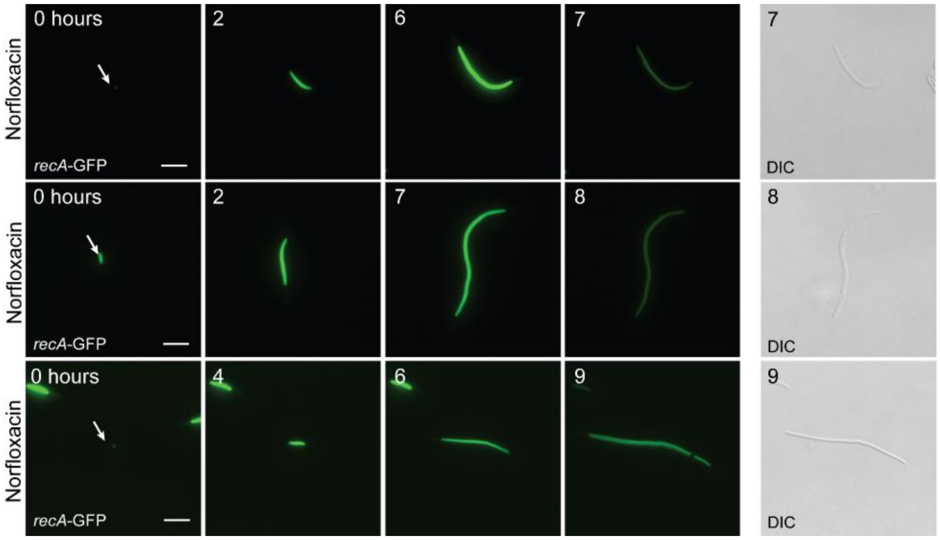

Appendix Figure S27. Representative micrographs of "failed" persisters carrying *recA*-GFP reporter after 5 µg/mL norfloxacin treatment. Scale and time are indicated on images. Scale bar: 8 µm.

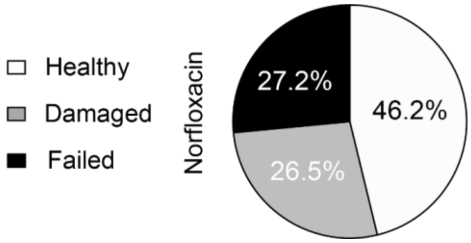

Appendix Figure S28. Pie chart depicting the proportion of resuscitated MG1655 persisters that are healthy, damaged, or fail after 10 µg/mL norfloxacin treatment.

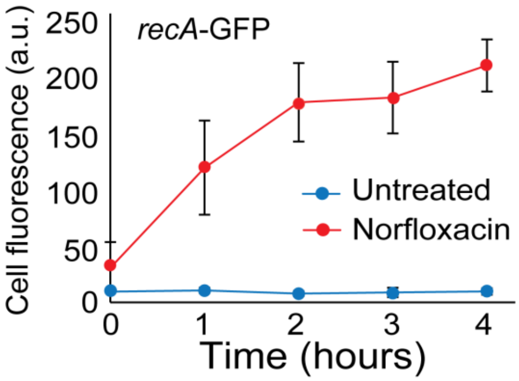

Appendix Figure S29. Norfloxacin induces *recA* expression in MG1655. Expression of *recA*-GFP reporter in individual MG1655 cell undergoing norfloxacin treatment compared with that of untreated cell. Values represent mean ± SD, n=10, biological replicates.

Deleted: Cellular fluoresce
after treatment with 2 μg/m
treatment. Transient *recA* i
from a single ampicillin per
intensity over time for micro
MG1655 ampicillin persiste
stationary phase cells. Val
n=10.¶

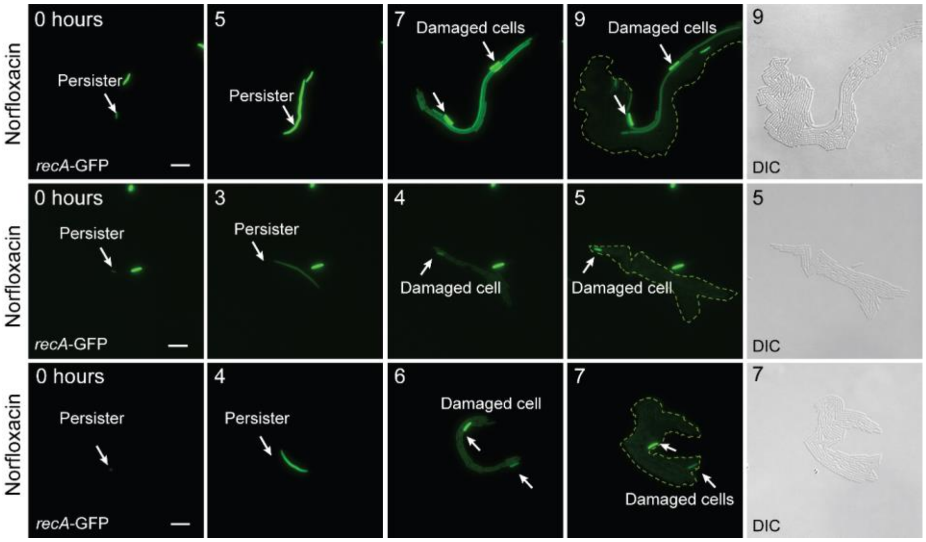

Appendix Figure S30. Representative micrographs of *recA* induction and persister partitioning after 5 μg/mL norfloxacin treatment. Example time courses of resuscitating persisters carrying a *recA*-GFP transcriptional reporter. Scale and time are indicated on images. Dashed line indicates microcolony boundary. Images of damaged persister (upper panel) are used in Figure 7A. Scale bar: 8 μm.

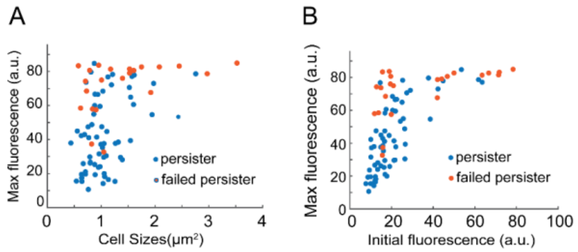

Appendix Figure S31. Comparing damaged and failed persisters after 5 μg/mL norfloxacin treatment. **(A)** Max cell fluorescence (*recA*:GFP) of individual norfloxacin persister or failed persister as a function of cell size during resuscitation. persister: n=63, 'failed' persister: n=23. **(B)** Maximum cell fluorescence (*recA*-GFP) of individual norfloxacin persister or failed persister as a function of initial cell fluorescence during resuscitation. persister: n=63, 'failed' persister: n=23.

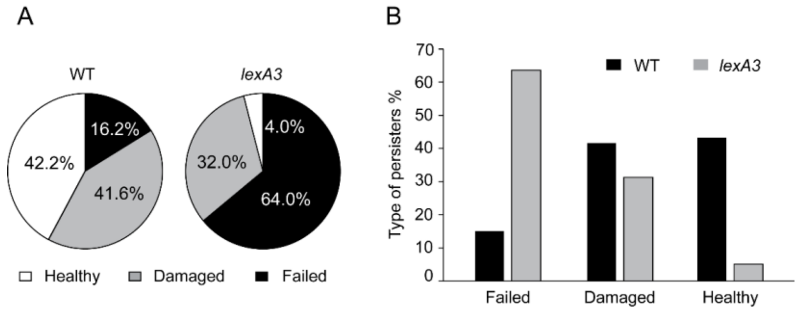

Appendix Figure S32. *lexA3* mutants produced higher fraction of failed persister after norfloxacin treatment. **(A)** Pie chart depicting the proportion of resuscitated persisters that are healthy, damaged, or fail after 5 μg/mL norfloxacin treatment. Left: wild-type MG1655; Right: *lexA3* mutant. **(B)** Column chart depicting the proportion of resuscitated persisters that are healthy, damaged, or fail after 5 μg/mL norfloxacin treatment. Black column: wild-type MG1655; Grey column: *lexA3* mutant. The same pie chart for wild-type control is used in Figure 6B.

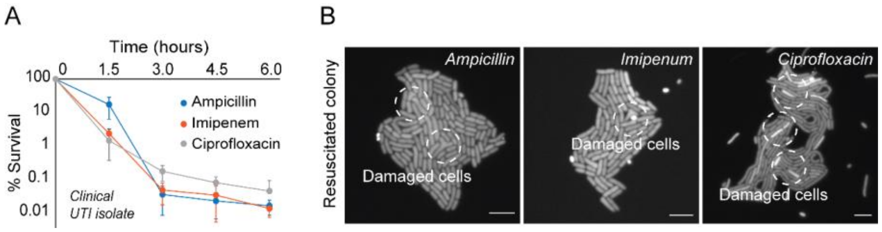

Appendix Figure S33: Persister partitioning during resuscitation in an *E. coli* strain isolated from a urinary tract infection. **(A)** Stationary phase UTI-isolate *E. coli* carrying pUA66 *pompC::gfp* reporter were inoculated into fresh LB and treated with 100 µg/mL ampicillin, 20 µg/mL imipenem, or 5 µg/mL ciprofloxacin (n=3, biological replicates). **(B)** Illustrative example microcolonies each deriving from a single resuscitated persister. Dashed circles indicate partitioned damaged cells. Antibiotic and scale are indicated on micrographs. Scale bar: 6 µm.

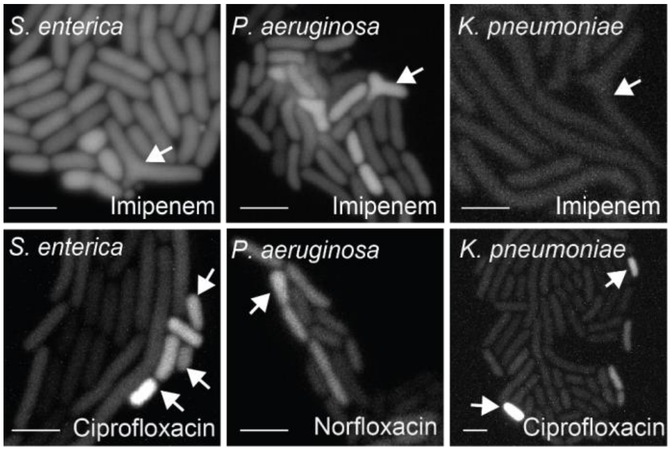

Appendix Figure S34: Persister partitioning in gram-negative pathogens. Example microcolonies deriving from single resuscitated persister cells of *S. enterica*, *P. aeruginosa* and *K. pneumonia* after treatment with imipenem or quinolone antibiotics. White arrows indicate persister with defects. Scale bar: 3 µm.

Additional References

Baba T, Ara T, Hasegawa M, Takai Y, Okumura Y, Baba M, Datsenko KA, Tomita M, Wanner BL, Mori H (2006) Construction of Escherichia coli K-12 in-frame, single-gene knockout mutants: the Keio collection. *Mol Syst Biol* 2: 2006 0008

Balleza E, Kim JM, Cluzel P (2018) Systematic characterization of maturation time of fluorescent proteins in living cells. *Nat Methods* 15: 47-51

Datsenko KA, Wanner BL (2000) One-step inactivation of chromosomal genes in Escherichia coli K-12 using PCR products. *Proc Natl Acad Sci U S A* 97: 6640-6645

Korch SB, Henderson TA, Hill TM (2003) Characterization of the hipA7 allele of Escherichia coli and evidence that high persistence is governed by (p)ppGpp synthesis. *Mol Microbiol* 50: 1199-1213

Pestrak MJ, Chaney SB, Eggleston HC, Dellos-Nolan S, Dixit S, Mathew-Steiner SS, Roy S, Parsek MR, Sen CK, Wozniak DJ (2018) Pseudomonas aeruginosa rugose small-colony variants evade host clearance, are hyper-inflammatory, and persist in multiple host environments. *PLoS Pathog* 14: e1006842

Zaslaver A, Bren A, Ronen M, Itzkovitz S, Kikoin I, Shavit S, Liebermeister W, Surette MG, Alon U (2006) A comprehensive library of fluorescent transcriptional reporters for Escherichia coli. *Nat Methods* 3: 623-628
